# PRR5, 7 and 9 positively modulate TOR signaling-mediated root cell proliferation by repressing *TANDEM ZINC FINGER 1* in *Arabidopsis*

**DOI:** 10.1093/nar/gkz191

**Published:** 2019-03-20

**Authors:** Bin Li, Yan Wang, Yuanyuan Zhang, Wenwen Tian, Kang Chong, Jyan-Chyun Jang, Lei Wang

**Affiliations:** 1 Key Laboratory of Plant Molecular Physiology, CAS Center for Excellence in Molecular Plant Sciences, Institute of Botany, Chinese Academy of Sciences, Beijing 10093, People's Republic of China; 2 University of Chinese Academy of Sciences; 3 Department of Horticulture and Crop Science, The Ohio State University, Columbus, OH 43210, USA; 4 Department of Molecular Genetics, The Ohio State University, Columbus, OH 43210, USA

## Abstract

Circadian clock coordinates numerous plant growth and developmental processes including cell elongation in the hypocotyl, whether or not it modulates cell proliferation is largely unknown. Here we have found that Pseudo Response Regulators (PRRs), essential components of circadian core oscillators, affect root meristem cell proliferation mediated by Target Of Rapamycin (TOR) signaling. The null mutants of PRRs display much reduced sensitivities to sugar-activated TOR signaling. We have subsequently identified *Tandem Zinc Finger 1*, encoding a processing body localized RNA-binding protein, as a direct target repressed by PRRs in mediating TOR signaling. Multiple lines of biochemical and genetic evidence have demonstrated that TZF1 acts downstream of PRRs to attenuate TOR signaling. Furthermore, TZF1 could directly bind *TOR* mRNA via its tandem zinc finger motif to affect *TOR* mRNA stability. Our findings support a notion that PRR-TZF1-TOR molecular axis modulates root meristem cell proliferation by integrating both transcriptional and post-transcriptional regulatory mechanisms.

## INTRODUCTION

Circadian clocks are ubiquitous biochemical time-keeping machineries that consist of non-orthologous interlocking feedback loops in distinct organisms (1). Time-keeping provides an adaptive advantage to higher plants by transducing the recurring daily changes of environmental cues to coordinate numerous physiological outputs, such as flowering time, biotic and abiotic stress responses, and homeostasis of cellular metabolism (1,2). In *Arabidopsis*, Pseudo Response Regulators (PRRs) are essential components of central oscillators that belong to a small gene family with five members. PRRs are characterized by a Pseudo-Receiver (PR) domain at the N-terminus and a CONSTANS, CONSTANS-LIKE and TOC1 (CCT) motif at the C-terminus (2,3). Besides the essential roles within core oscillators, PRR5, PRR7, together with PRR9 play pivotal roles in coordinating many daily cycling physiological processes by timing the expression of numerous downstream transcription factors, such as those involved in oxidative stress response and stomata opening (4,5). Interestingly, the tricarboxylic acid (TCA) cycle intermediates are significantly increased in *prr5 prr7 prr9* triple mutant (hereafter as *prr579*) (6). The *prr579* triple mutant also contains unusually high levels of starch in both sink and source leaves, especially at dawn when starch turnover rate is low (7). Recently, it has been revealed that PRR7 acts as an entry point for circadian entrainment mediated by photosynthesized sugars (8). Furthermore, the high chlorophyll contents and elevated glucose accumulation at dawn in *prr5, prr7* and *prr9* resemble that in a glucose sensor (Hexokinase 1, HXK1) mutant *glucose insensitive 2* (*gin2*) (8), thus raising a possibility that PRR proteins are closely involved in sugar signaling. However, whether PRR proteins are also involved in Target Of Rapamycin (TOR) signaling, which is a major nutrient and energy sensing mechanism in higher plants (9), remains unclear. Nevertheless, given there is a close connection between circadian clock and TOR signaling in mammals (10–12), it is conceivable that PRR proteins might mediate this much-needed crosstalk in higher plants.

TOR protein is a highly conserved large phosphatidylinositol 3-kinase-like protein kinase (PIKK), affecting plant root growth by integrating phytohormone and nutrient signals to modulate cell proliferation in the root meristem (13–16). The TOR kinase is present in two distinct complexes in mammals, namely TORC1 and TORC2, while only the TORC1 components are conserved in plants (17–19). TORC1 is composed of TOR, Regulatory Associated Protein of mTOR (RAPTOR), and the Lethal with SEC13 protein 8 (LST8) (17). The TOR signaling pathway governs a myriad of cellular processes, such as protein translation, ribosome biogenesis, and starch and triacylglycerol accumulation (9,13–15). The null mutant of the *Arabidopsis TOR* gene is embryo-lethal (20,21), reinforcing its essential role in plant growth and development. TOR activation can promote root cell proliferation via direct phosphorylation of E2Fa, which in turn enhances S-phase genes expression at the transcriptional level (9,16). Glucose-TOR signaling also directs the expression of numerous genes mainly involved in defense response, adaptation, and survival in plants (9). Other notable components of the plant TOR signaling include Type 2A-phosphatase-associated protein 46 kD (Tap46) (22), ribosomal protein S6 (RPS6) (23), and possibly Tandem Zinc Finger 1 (TZF1) (9,24).

The *Arabidopsis* TZF1 and 10 other family members belong to the subfamily IX of AtC3H family of Tandem CCCH Zinc Finger proteins (TZFs), and they share a conserved arginine-rich-TZF motif (RR-TZF) (25–27). In higher plants, TZFs are involved in hormone- and environmental cues-mediated plant growth and stress responses with elusive molecular mechanisms (28,29). The nucleo-cytoplasmic shuttling protein TZF1 is primarily localized in Processing Bodies (PBs) and Stress Granules (SGs), which are aggregations of cytoplasmic messenger ribonucleoprotein complexes involved in post-transcriptional regulation of gene expression (30). *TZF1* overexpression plants (hereafter as *TZF1 OE*) are compact, extremely late flowering, and abiotic stress tolerant, whereas its null mutants have no distinct phenotypes due to functional redundancy with other family members (31). The integrity of the TZF motif is required for TZF1 to target and degrade AU-rich element-containing mRNAs (26). TZF1 has been implicated as a potential component in TOR signaling, however, its role in the TOR signaling is unknown (16,24).

Here, we have uncovered a close intersection between PRR proteins and TOR signaling in modulating root architecture via the control of root meristem cell proliferation. The *prr579* mutant is defective in glucose-activated TOR signaling with reduced cell proliferation activity in the root meristem. Physiological, biochemical and genetic analysis revealed that *TZF1* is a direct target of PRR5, PRR7 and PRR9 proteins in mediating the TOR signaling. We have further demonstrated that TZF1 directly binds *TOR* mRNA through its tandem zinc finger motif and affects *TOR* mRNA stability. As the interconnection between circadian clock and TOR signaling has also been discovered in flies and mice, our findings thus revealed an evolutionarily conserved regulatory link between circadian clock components and TOR signaling pathway in higher plants, which could shape root architecture by integrating a novel circadian output with endogenous energy status.

## MATERIALS AND METHODS

### Plant materials and plasmid construction

The *Arabidopsis thaliana* Columbia-0 (Col-0), *prr5–11 prr7–11 prr9–10* mutant (32), and transgenic line *TZF1 OE* (31) were used in this study. To generate *TZF1 OE prr579* line, *TZF1 OE* was crossed to *prr579* and the homozygote was screened by PCR genotyping. To obtain loss-of-function *TZF1 OE* alleles, seeds of *TZF1 OE* homozygous plant were treated with ethyl methanesulfonate, followed by a screen aiming to identify M2 revertants that lost the typical compact and late flowering phenotypes of *TZF1 OE* plants. Non-segregating M3 alleles were then identified by Sanger sequencing. Overexpression lines of *TZF4, TZF5* and *TZF6* were generated as described (33), and overexpression lines of *TZF2* and *TZF3* were generated with similar methods. The growth conditions used in the experiments were LD (12-h light/12-h dark, white light, 22°C), LL (constant white light, 22°C), DD (constant darkness, 22°C) as indicated. The light intensity was 48 μmol m^−1^ s^−1^ except otherwise stated.

For glucose-induced reactivation of root growth assay, the seeds were sterilized and incubated in sugar-free liquid medium (half strength of Murashige and Skoog medium (1/2 MS) without vitamins) (Phytotech), pH 5.7, for 2 days at 4°C, and then germinated in weak light (13 μmol m^−1^ s^−1^, 12-h light/12-h dark, 22°C) for 3 days to enter the mitotically quiescent state, as described previously (9). Quiescent seedlings were then transferred to a medium (1/2 MS, pH 5.7) containing either 15 mM glucose or 15 mM sucrose to grow for additional 3 days in weak light (13 μmol m^−1^ s^−1^, 12-h light/12-h dark, 22°C) before root meristem reactivation and root growth analyses. Root length was measured by using NIH ImageJ software. One-way ANOVA was used for the statistical analysis by SPSS software.

To determine the root architecture, the seeds of Col-0, *prr579* and *TZF1 OE* were sterilized, placed on MS plates (0.8% agar, pH 5.7) containing 1% mannitol, glucose or sucrose for 2 days at 4°C, and then incubated in weak light (13 μmol m^−1^ s^−1^, LD, 22°C) for 2 weeks to observe root growth. Root length was measured by using NIH ImageJ software. One-way ANOVA was used for the statistical analysis by SPSS software.

For lateral root analysis, the seeds of Col-0, *prr579* and *TZF1 OE* were sterilized, placed on MS plates (with 0.8% agar, pH 5.7) containing 1% sucrose for 2 days at 4°C, and then cultured at 22°C under LD conditions for 2 weeks to observe lateral root growth. *t*-test was used for the statistical analysis by SPSS software.

For transient transformation experiment in *N. benthamiana*, an 1,606 bp *TZF1* promoter was amplified from Col-0 genomic DNA and inserted into promotor-less *LUC-N-1300* vector through *EcoR*I and *Kpn*I to generate the *TZF1pro:LUC*-N-1300 reporter construct. The effector plasmids of GFP-PRRs were made as described (32). To make a *TZF1pro:LUC* for protoplast transient expression assay in *Arabidopsis*, the same *TZF1* promoter fragment was digested with *Bgl*II and *Bsu36*I and subcloned into promotor-less *pLUC-999*. Point-mutation of TZF1 effectors used in *Arabidopsis* protoplast transient gene expression analysis were generated by replacing the wild-type *TZF1* in the *pGEX-KG* vector, whereas the reporter constructs were as described (26). The primers used for making aforementioned constructs are listed in Supplementary Table S1.

### RNA extraction and qPCR analysis

Total mRNA was extracted using TRIZOL reagent (Invitrogen) following manufacturer's instructions. RNA quantification and purity assessment were determined by Thermo Scientific NanoDrop spectrophotometer 2000 (NanoDrop Technologies), after DNase I digestion. Equal amount of RNA was used for reverse transcription. For reverse transcription, 1 μg of DNase (DNA-free™ DNA Removal Kit, Invitrogen) digested RNA was used for reverse transcription by M-MLV reverse transcriptase (Promega) in 20 μl reverse transcription reaction with the primer of OligodT_18_. The qPCR was performed using the Real Master Mix (SYBR Green I) (TOYOBO) on the Mx3000P real-time PCR system. The qPCR reaction was performed as follows: 95°C for 2 min, 40 cycles of 95°C for 15 s, 55°C for 15 s, and 72°C for 15 s, followed with disassociation curve analysis: 95°C for 15 s, 55°C for 30 s, and 95°C for 30 s. Collected the data using the MxPro-Mx3000P real-time PCR system, and the 2^−△CT^ method was used to calculate the gene expression level. To analyze the gene expression level, the geometric mean of *ACT2* and *TUB4* expression were used as a normalization control. The primers for qPCR analysis were listed in Supplementary Table S1.

### EdU staining and confocal microscopy

EdU staining was performed as described (9,34). Briefly, the seeds were sterilized, placed in sugar-free liquid medium (pH 5.7, adjusted with KOH) for 2 days at 4°C, and then germinated in weak light (13 μmol m^−1^ s^−1^, LD, 22°C) for 3 days to enter the mitotically quiescent state. Quiescent seedlings were transferred into a medium (1/2MS, pH 5.7) containing 15 mM glucose or 15 mM sucrose for 1 day before EdU staining was conducted. The seedlings were stained with 1 μM EdU for 30 min. The roots were dissected and treated with 3.7% formaldehyde solution in PBS solution with 0.1% Triton X-100 for 30 min. After removing the fixative, the roots were washed three times with PBS solution (10-min each). The roots were then incubated in EdU detection cocktail (Invitrogen) for 30 minutes at room temperature in the dark, followed by PBS solution washing three times (10-min each). The root meristem cells were observed using a confocal laser scanning microscope (Leica TCS SP5).

### ChIP-qPCR assay

The 10-day-old seedlings of Col-0, *PRR5pro:PRR5-GFP, PRR7pro:PRR7-GFP, PRR9pro:PRR9-GFP* grown at 22°C on MS plates with 1% sucrose and 0.7% agar under LD conditions were harvested for chromatin immunoprecipitation after formaldehyde cross-linking. The chromatin sample was precleared with 20 μl of salmon sperm-sheared DNA/protein Agarose beads (Millipore) for 1 h at 4°C. For each immunoprecipitation, 40 μl of salmon sperm-sheared DNA/protein Agarose beads and 0.8 μg GFP antibody (Invitrogen, A11120) were used. The immunoprecipitation was performed at 4°C overnight with gentle agitation. The beads were sequentially washed by low salt wash buffer, high salt wash buffer, and LiCl buffer once, and the TE buffer twice. The protein-DNA complexes were eluted from beads with elution buffer at 65°C for 15 min. To reverse the cross-linking, 20 μl of 5 M NaCl was added to the eluted solution and incubated at 65°C overnight. Precipitated DNA was extracted and then qPCR was performed to determine the abundance of target genes. The primers used in this study are list in Supplementary Table S1. The qPCR was performed using the Real Master Mix (SYBR Green I) (TOYOBO) on the Mx3000P real-time PCR system. The 2^−△CT^ method was used to calculate the ratio of IP to the input, and then normalized with Col-0 among respective biological replicate. The *t*-test was conducted for the comparison between *PRRn:PRRn-GFP* and Col-0 from three individual biological replicates.

### Transient gene expression analysis

Protoplasts were isolated from the rosette leaves of 4-week-old *Arabidopsis* plants growing in LD conditions, as previously described (32). For transient gene expression analysis, 200 μl of protoplasts were transferred to a 2 ml microfuge tube containing 6 μg effector plasmid plus 2 μg reporter plasmid and 2 μg of *35S:GUS* plasmid as internal control. The protoplasts were incubated for 16 h at 22°C. The luciferase assay system (Promega) was used for luminescence measurements on GloMax 20/20 luminometer. The GUS activity was detected by MUG substrate (Alfa) on GloMax 20/20 luminometer.

For transient gene expression analysis in *N. benthamiana, Agrobacterium tumefaciens* AGL carrying various constructs (*TZF1pro:LUC-N-1300, CCA1pro:LUC, 35S:GFP-PRR9, 35S:GFP-PRR7, 35S:GFP-PRR5, 35S:GFP-blank*) were cultured overnight at 28°C. *TZF1pro:LUC-N-1300* and *CCA1pro:LUC* were used as reporters, and *35S:GFP-PRR9, 35S:GFP-PRR7, 35S:GFP-PRR5*, and *35S:GFP-blank* were used as effectors. Cultured *Agrobacterium* cells were collected and resuspended in an infiltration solution (containing 10 mM MgCl_2_, 10 mM MES, and 1 mM Acetosyringone). The leaves of 6-week-old *N. benthamiana* grown in 25°C 12 h light/12 h dark cycles were used for infiltration analysis. The level of gene expression was determined by the luminescence intensity derived from luciferase activity using a CCD camera (LN/1300-EB/1, Princeton Instruments) 2 days after infiltration.

### RNA immunoprecipitation (RIP) and RT-qPCR analysis

RIP was performed as described (35). Briefly, 10-day-old seedlings of Col-0, *TZF1 OE*, *TZF1(G155E) OE* and *TZF1(H186Y) OE* grown in MS medium with 1% sucrose at 22°C under LD conditions were harvested at ZT8 for crosslinking. The cross-linked tissues (1.5 g) were ground with liquid nitrogen and resuspended in 60°C prewarmed 750 μl RIP lysis buffer to make viscous homogenates. After centrifugation, the cell extract supernatant was filtered through a 0.45 μm filter. The extract was pre-cleared twice with 50 μl Sepharose beads before mixing with 15 μl washed Sepharose beads with GFP antibody (Invitrogen, A11120). The immunoprecipitation was performed at 4°C for 2 h followed by washing the beads with RIP washing buffer for 10 min at 4°C three times. The beads were then washed with RIP lysis buffer for 5 min at 4°C. The RNA was purified from the immunoprecipitated RNPs and 100 μl input, respectively, using the TRIzol^®^ reagent (Invitrogen). For quantitative analysis of TZF1-binding RNA, reverse transcription was conducted using RETROscript^®^ Kit. After DNase treatment, RNA samples were reverse transcribed with random hexamer primers. Quantitative PCR was performed to determine the level of TZF1-bound RNA, and the 2^−△CT^ method was used to calculate the ratio of RIP to the input, and then normalized with Col-0 among respective biological replicate.

### Bioluminescence assays and circadian rhythm analysis

To generate *TZF1 OE CCA1pro:LUC* line, *TZF1 OE* was crossed to *CCA1pro:LUC* reporter line. The homozygote was screened through positive luminescence and kanamycin resistant phenotypes. Seven-days-old seedlings grown at 22°C on MS plates contained 3% sucrose and 0.7% agar in 12-h light/12-h dark photoperiod were transferred to constant red light at 22°C and sprayed with luciferin for the measurement of the luminescence using a CCD camera (LN/1300-EB/1, Princeton Instruments). Luminescence images were processed and quantified by MetaMorph software. Data were imported into the Biological Rhythms Analysis software system (BRASS v2.14 (36), available from www.amillar.org) and analyzed with the Fourier transform-nonlinear least-squares suite of programs. Period lengths were reported as variance-weighted periods ± s.e.m., which were estimated using bioluminescence data with a time window from 24 to 144 h.

## RESULTS

### Glc-TOR signaling mediated cell proliferation is compromised in *prr579* mutant

**Figure 1.**
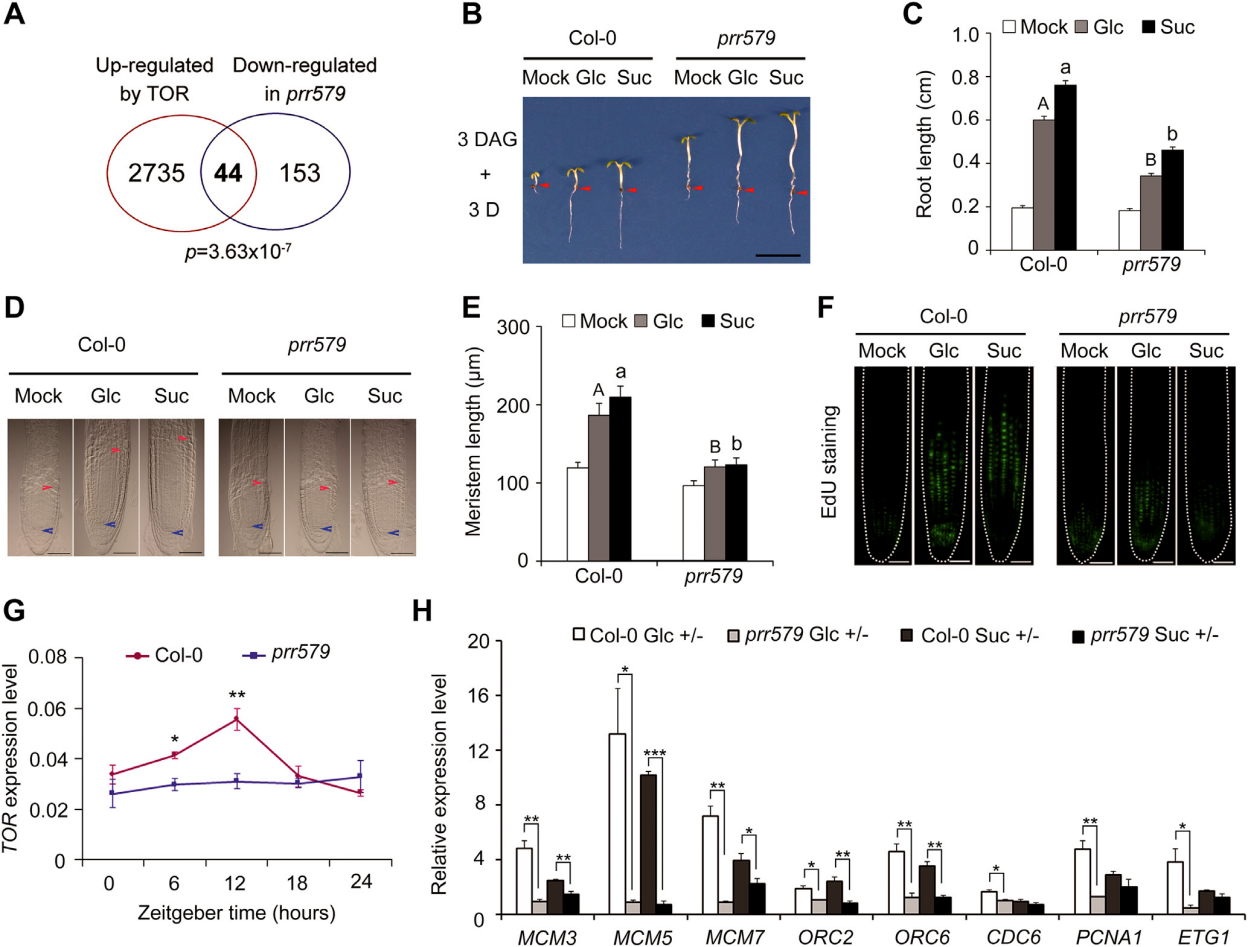
Glc-TOR signaling is compromised in *prr579* mutant. (**A**) Venn digram showing overlap between genes activated by TOR signaling (9) and genes down regulated in *prr579* mutant (39). (**B**) Compared to the WT (Col-0), primary root growth was reduced in *prr579* after sugar-induced reactivation of TOR-signaling. Three-day-old seedlings grown in liquid sugar-free 1/2 MS were treated with 15 mM Glc or Suc for 3 days in weak light (22°C, LD). Red arrows indicate the hypocotyl and root junctions. DAG, days after germination; D, day; Mock, sugar-free; Glc, glucose; Suc, sucrose; (scale bar: 0.5 cm). (**C**) Quantitative analysis of primary root length in (**B**). Data represent mean ± s.e.m. (*n* = 18). The experiment was carried out with three biological replicates with similar results. The lengths of primary roots were measured by using ImageJ. (D, E) Root meristem proliferation was not fully activated in *prr579* mutant after activation of TOR signaling by sugars. (**D**) Differential interference contrast (DIC) imaging of root meristems (scale bars: 50 μm). The blue arrow indicates the root quiescent cells, and the red arrow indicates the transition between meristem zone and elongation zone. (**E**) Quantitative analysis of root meristem length in (**D**). Data represent mean ± s.e.m. (*n* = 13). The lengths of root meristems were measured by using ImageJ. (**F**) The *prr579* mutant was defective in S-phase entry of cell cycle as evidenced by reduced 5-ethynyl-2′-deoxyuridine staining (EdU) signals *in situ* (scale bars: 50 μm). (**G**) Time-course expression of *TOR* in Col-0 and *prr579*. The total RNA was isolated from the roots of 6-day-old seedlings grown in half-strength liquid MS medium without sugar in 12-h weak light (13 μmol m^−1^ s^−1^)/12-h dark cycles at 22°C. The gene expression level was normalized by the geometric mean of *ACT2* and *TUB4*expression. Data represent mean ± s.e.m. of three biological replicates. (**H**) Expression of S-phase marker genes was reduced in *prr579* mutant. For RNA extraction, the roots were harvested from the seedlings treated with 15 mM Glc or Suc for 3 days after growing in liquid sugar-free 1/2 MS for 3 days in weak light (22°C, LD) at ZT8. The gene expression level was normalized by *ACT2* expression. The bar values represent the gene expression levels in sugar treatments relative to the corresponding expression levels in sugar-free condition. Data represent mean ± s.e.m. of three biological repeats. Different letters in (C) and (E) represent significant difference at *P* < 0.01 by one-way ANOVA using SPSS software. Uppercase letters compare with each other in 15 mM Glc treatment condition, and lowercase letters compare with each other in 15 mM Suc treatment condition. The asterisk in (**G**) and (**H**) indicates significant difference as * *P* < 0.05, ** *P* < 0.01, and *** *P* < 0.001 by *t*-test.

The *prr579* mutant displayed a number of hallmarks of TOR signaling deficiency, such as accumulation of abnormally high levels of starch and TCA cycle intermediates (6,7,37,38). Importantly, the mRNA level of *TOR* displayed a clear oscillation pattern in both light/dark and continuous light conditions (Supplementary Figure S1). Moreover, ∼22% (44/197) genes with decreased abundance in *prr579* mutant reported in previously microarray analysis (39) were overlapped with TOR activated genes (*p* = 3.63 × 10^−7^, hypergeometric probability test) (Figure 1A, Supplementary Table S2), further indicating that TOR signaling might be defective in *prr579* mutant. Hence, we were prompted to examine whether *prr579* mutant had abnormal responses to TOR signaling using a well-established method (9). After endogenous sugar depletion by growing germinated seeds under photosynthesis-constrained weak light condition for three days, the treated seedlings were transferred to media containing either glucose or sucrose to reactivate the arrested root meristems by Glc-TOR mediated transcriptional reprogramming (9). Compared to the WT, the primary roots of *prr579* mutant were significantly shortened, accompanied by reduced quiescent root meristems (Figure 1B–E). In contrast, the hypocotyls of *prr579* mutant were significantly longer than that in Col-0, regardless of sugar treatments (Figure 1B), implicating cell proliferation in the root meristems was specifically inhibited by attenuated Glc-TOR signaling. The 5-Ethynyl-2′-deoxyuridine (EdU) staining was used as an indicator for the activity of cell cycle S-phase entry (9). Compared to the WT, EdU staining signals in *prr579* mutant were also significantly reduced in the presence of glucose or sucrose (Figure 1F). These results supported the idea that Glc-TOR signaling in quiescent root meristems was compromised in *prr579* mutant. TOR-signaling marker genes were then examined to determine whether changes in transcript levels were consistent with the phenotypes. Compared to the WT, the transcript level and amplitude of *TOR* were significantly dampened in *prr579* in a time course experiment (Figure 1G). Accordingly, transcript levels of a subset of Glc-TOR downstream target genes, including *ORC2/6 (ORIGIN RECOGNITION COMPLEX), MCM3/5/7 (MINOCHROMOSOME MAINTENANCE), CDC6 (CELL DIVISION CYCLE), ETG1 (E2F TARGET GENE)* and *PCNA1 (PROLIFERATING CELL NUCLEAR ANTIGEN)* (9), were significantly reduced in *prr579* mutant as well (Figure 1H). Notably, *PRR5, PRR7* and *PRR9* appeared to redundantly affect Glc-TOR signaling, as the corresponding single mutants were all less sensitive to Glc-TOR signaling compared with Col-0 (Supplementary Figure S2). Furthermore, overexpression of *PRR9* using a constitutive promoter resulted in elevated sensitivity to Glc-TOR signaling, in concomitance with enhanced expression of *TOR* (Supplementary Figure S3A–C), further implicating that PRR proteins were potential modulators of Glc-TOR signaling.

### *TZF1* is a direct target gene of PRR proteins in mediating Glc-TOR signaling

**Figure 2.**
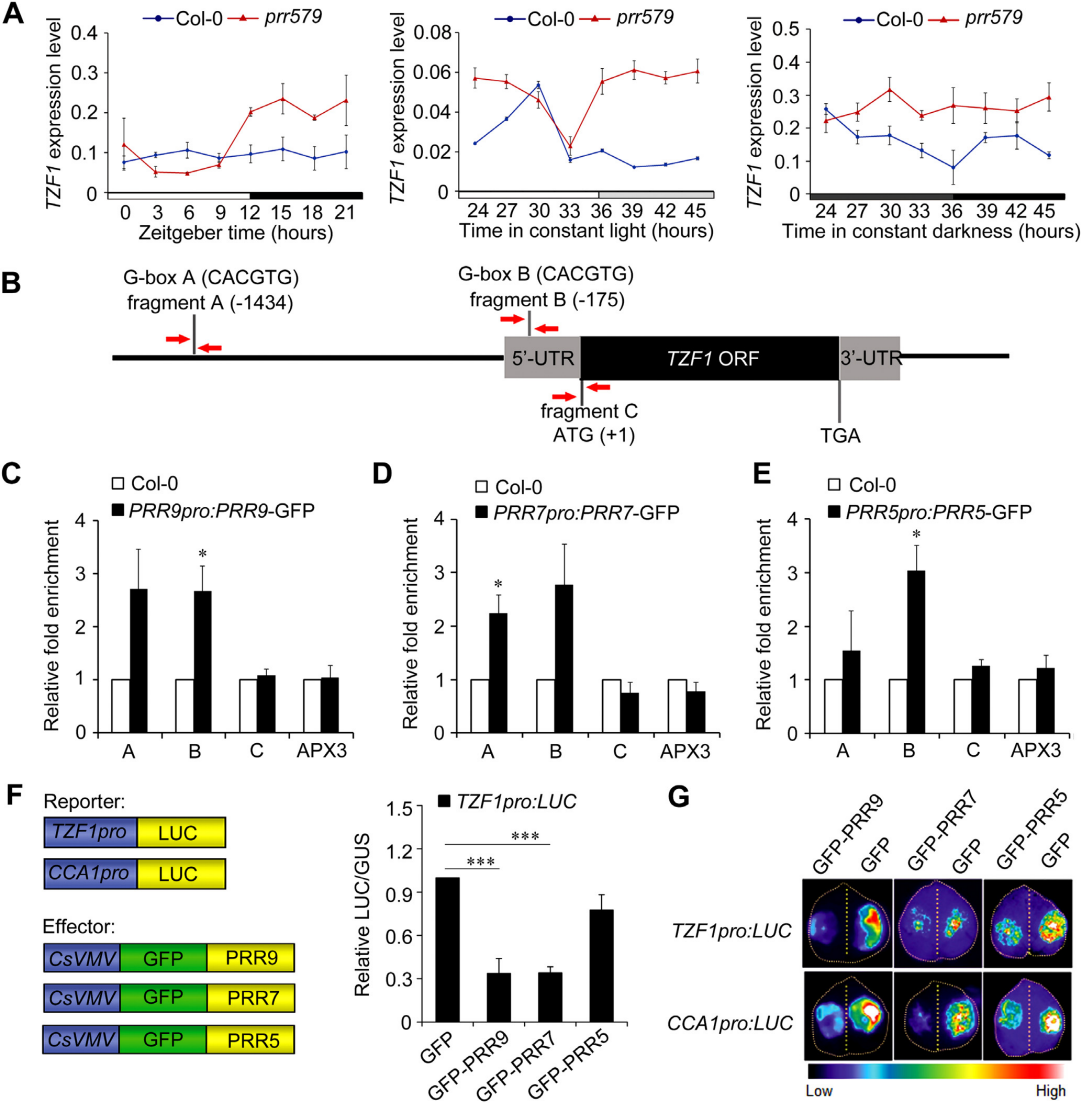
*TZF1* is a direct transcriptional target of PRR5, 7 and 9. (**A**) RT-qPCR analysis of *TZF1* expression in Col-0 and *prr579* mutant. Ten-day-old seedlings grown in LD condition were transferred to the LL condition or DD condition. The samples were collected every 3 hours over a 24-h time-course. The gene expression level was normalized by the geometric mean of *ACT2* and *TUB4* expression. Data represent mean ± s.e.m. of three biological repeats. (**B**) Diagram depicting *TZF1* gene structure and the potential PRR proteins binding G-box elements in *TZF1* promoter. Red arrows indicate the locations of oligo primers used for ChIP-qPCR analysis. (**C**-**E**) ChIP-qPCR results showing enriched DNA fragments containing G-box element. Ten-day-old seedlings of Col-0, *PRR5pro:PRR5-GFP* (ZT10), *PRR7pro:PRR7-GFP* (ZT8), *PRR9pro:PRR9-GFP* (ZT4) grown at 22°C under LD condition were used for chromatin immunoprecipitation. Error bars represent standard error of three biological replicates. The asterisk indicates significant difference relative to Col-0 at * *P* < 0.05 by *t*-test. The amplicon of *APX3* promoter was used as a negative control. (**F**) *TZF1* expression was repressed by PRR proteins in protoplast transient expression analyses. Data represent mean ± s.e.m. *GUS* expression was used as transformation control to normalize the *LUC* expression from the reporter. Error bars represent standard errors of three technical replicates. The result was shown from one of three independent experiments with similar results. The asterisk indicates significant difference as *** *P* < 0.001 by *t*-test. (**G**) Transient expression analysis using *N. benthamiana* leaf infiltration showing that *TZF1* expression was repressed by PRR proteins. *CCA1pro:LUC* was used as a positive control for the repression by PRRs. The result was shown from one of three independent experiments with similar results.

PRR5, PRR7, and PRR9 can act as transcription repressors by recruiting TOPLESS family protein and HDA6/HDA19 (histone deacetylase 6/19) to form a tripartite repression complex (32). *TOR* is unlikely a direct target gene of PRR5/7/9, as its transcript level is lower in *prr579* mutant than that in the WT. To identify downstream components of PRR proteins in mediating Glc-TOR signaling, we analyzed the public available transcriptome data of the *prr579* mutant (39). The mRNA abundance of *TZF1*, a potential component in TOR signaling pathway (9,24), was increased ∼2.8-fold in *prr579* mutant (39). The transcript level of *TZF1* in *prr579* mutant was further investigated in time-course experiments under light/dark, constant light, and constant darkness conditions. As expected, the *TZF1* mRNA abundance was higher in *prr579* mutant in subjective day under constant light, and in all time points under constant dark (Figure 2A). Interestingly, the temporal expression pattern of *TZF1* peaked at subjective daytime while in trough level at subjective night (Supplementary Figure S4), consistent with the oscillation patterns of target genes of PRRs. Collectively, these results indicated that TZF1 might act as a downstream adaptor of PRR proteins in mediating TOR signaling. This led us to hypothesize that TOR signaling defect in *prr579* mutant might be due to elevated *TZF1* expression.

To test if *TZF1* were a direct downstream target gene of PRR proteins in mediating Glc-TOR signaling, we examined cis-acting elements of *TZF1* promoter between -1,434 bp and the start codon. Two canonical G-box (CACGTG) elements, which are required for PRRs-mediated transcriptional regulation (5,40,41), were found in this region (Figure 2B). To determine if PRR proteins could bind G-box regions in the *TZF1* promoter *in vivo*, we performed ChIP-qPCR using previously established transgenic lines of *PRRn:PRRn-GFP* (‘n’ stands for 5, 7 or 9) (42). The tissues were collected at the respective peak time of each PRR protein (42). Significant fold enrichment of G-box B region in the *TZF1* promoter was found for *PRR9* and *PRR5*, whil*e* significant fold enrichment of G-box A region was found for *PRR7* (Figure 2C–E). No evident enrichment was found for amplicon C that extended beyond the start codon of *TZF1* coding region or the negative control *APX3* promoter. For a positive control, we confirmed the binding of *CCA1* promoter by PRR5, PRR7 and PRR9 (Supplementary Figure S5) as previously reported (41). Next, we examined the direct repressive roles of PRR proteins on *TZF1* expression by conducting transient gene expression analysis using Arabidopsis protoplasts and *Nicotiana benthamiana* leaves, respectively. As shown in Figure 2F and G, ectopic expression of *PRR7* and *PRR9* significantly repressed the promoter activity of *TZF1*, while PRR5 marginally repressed *TZF1pro:LUC* in both assays, consistent with its weaker binding to *TZF1* promoter. Taken together, PRR proteins could differentially bind *TZF1* promoter and transcriptionally repress *TZF1* expression.

### TZF1 negatively modulates Glc-TOR signaling

**Figure 3.**
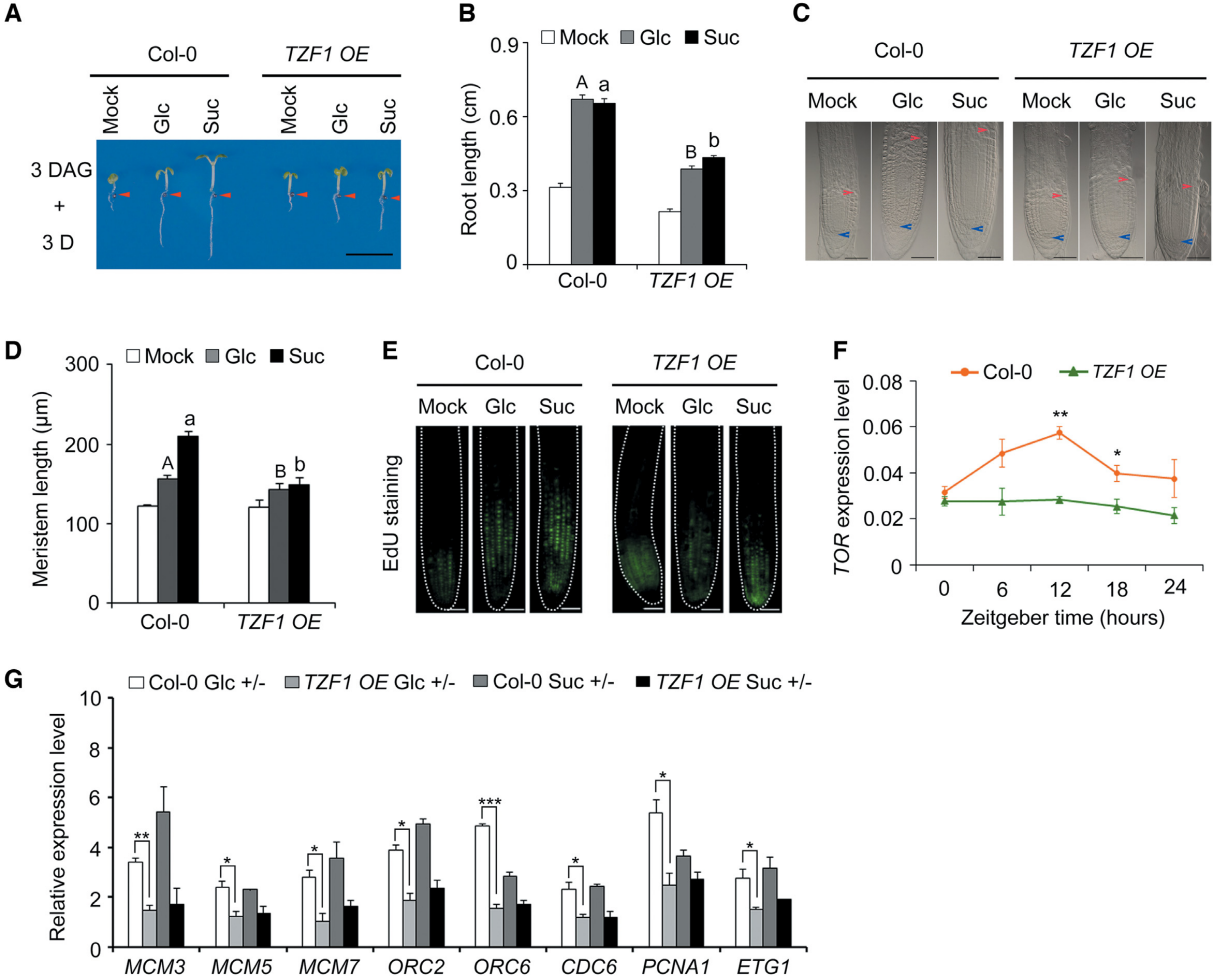
Glc-TOR signaling is compromised in *TZF1 OE* line. (**A**) The diminished activation of Glc-TOR signaling in *TZF1 OE* line was evidenced by reduced primary root elongation. Three-day-old seedlings grown in liquid sugar-free 1/2 MS were treated with 15 mM Glc or Suc for 3 days in weak light (22°C, LD). Red arrows indicate the hypocotyl and root junctions. DAG, days after germination; D, day; Mock, sugar-free; Glc, glucose; Suc, sucrose; (scale bar: 0.5 cm). (**B**) Quantitative analysis of primary root length in (A). Data represent mean ± s.e.m. of 18 plants. The result was shown from one of three independent experiments with similar results. Root lengths were measured by using ImageJ. (**C**) DIC imaging of the root meristem zones of Col-0 and *TZF1 OE* line (scale bars: 50 μm). The blue arrow indicates the root quiescent cells, and the red arrow indicates the transition between meristem zone and elongation zone. (**D**) Quantitative analysis of the root meristem size with or without sugar treatment. Data represent mean ± s.e.m. of 13 plants. The result was shown from one of three independent experiments with similar results. The root meristem size was measured by using ImageJ. (**E**) S-phase entry of cell cycle in primary roots is reduced in *TZF1 OE* as evidenced by reduced EdU staining signals *in situ* (scale bar: 50 μm). (**F**) Time-course expression of *TOR* in Col-0 and *TZF1 OE*. The gene expression level was normalized by the geometric mean of *ACT2* and *TUB4* expression. The total RNA was isolated from the roots of 6-day-old seedlings grown in half-strength liquid MS medium without sugar in 12-h weak light (13 μmol m^−1^ s^−1^)/12-h dark cycles at 22°C. Data represent mean ± s.e.m. of three biological replicates. (**G**) Expression of S-phase marker genes was reduced in *TZF1 OE* with sugar treatments. For RNA extraction, the roots were harvested from the seedlings treated with 15 mM Glc or Suc for 3 days after growing in liquid sugar-free 1/2 MS for 3 days in weak light (22°C, LD) at ZT8. The gene expression level was normalized by *ACT2* expression. The bar value represented the gene expression levels in sugars treatment relative to the corresponding expression levels in sugar-free condition. Data represent mean ± s.e.m. of three biological repeats. Different letters in (B) and (D) represent significant difference at *P* < 0.01 by one-way ANOVA using SPSS software. Uppercase letters compare with each other in 15 mM Glc treatment condition, and lowercase letters compare with each other in 15 mM Suc treatment condition. The asterisk in (F) and (G) indicates significant difference as * *P* < 0.05, ** *P* < 0.01, and *** *P* < 0.001 by *t*-test.

**Figure 4.**
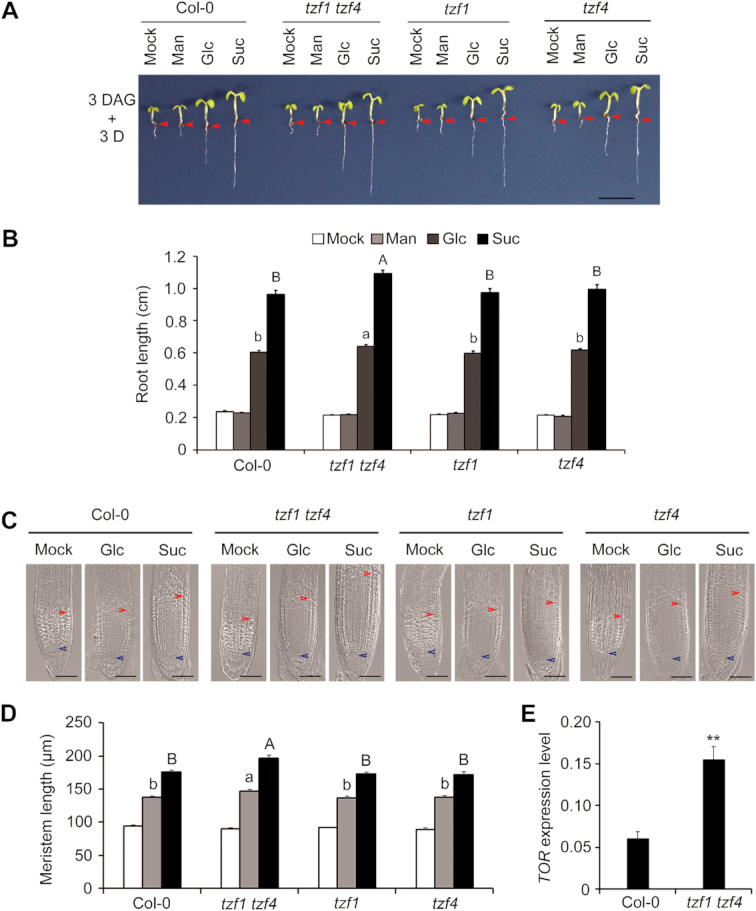
Functional redundancy of *TZF1* and *TZF4* in mediating Glc-TOR signaling. (**A**, **B**) The *tzf1 tzf4* double mutant displays a modest but significant synergistic enhancement of Glc-TOR signaling response. Red arrows indicate the hypocotyl and root junctions. DAG, days after germination; D, day; Mock, sugar-free; Man, mannitol; Glc, glucose; Suc, sucrose; scale bars: 0.5 cm. Data represent mean ± s.e.m. of 16 plants. The result was shown from one of the three biological replicates with similar results. Root lengths were measured by using ImageJ. (**C**) DIC imaging of root meristem zones. Scale bar: 50 μm. The blue arrow indicates the root quiescent cells, and the red arrow indicates the transition between root meristem zone and elongation zone. (**D**) Quantitative analysis of the root meristem size in (**C**). Data represent mean ± s.e.m. of 16 plants. The result was shown from one of three independent experiments with similar results. The root meristem size was measured by using ImageJ. Different letters in (**B**) and (**D**) indicate significant differences at *P* < 0.05 by one-way ANOVA. Uppercase letters compared with each other in 15 mM Glc treatment condition, and lowercase letters compared with each other in 15 mM Suc treatment condition. (**E**) *TOR* expression level is significantly enhanced in *tzf1 tzf4*. The total RNA was isolated from the roots of 6-day-old seedlings grown in half-strength liquid MS medium without sugar in 12-h weak light (13 μmol m^−1^ s^−1^) /12-h dark cycles at 22°C and harvested at ZT6. The gene expression level was normalized by the geometric mean of *ACT2* and *TUB4* expression. Data represent mean ± s.e.m. of three biological replicates. The asterisks indicate significant difference at *P* < 0.01 (**) by *t*-test.

Since *TZF1* null mutant was indistinguishable from the WT plants due to functional redundancy with other family members, we determined the effects of TZF1 on Glc-TOR signaling by using the *TZF1/2/3* RNAi lines with significant reduction of mRNA abundance of *TZF1, TZF2* and *TZF3* (31). However, the *TZF1/2/3* RNAi plants did not show obvious changes in Glc-TOR signaling (Supplementary Figure S6A and B), probably due to incomplete knockdown and functional redundancy with yet other TZF members. However, similar to *prr579* mutant, *TZF1 OE* plants displayed reduced sensitivities to Glc-TOR signaling, with shorter primary roots and reduced size of root meristems (Figure 3A–E, Supplementary Figure S7). This appeared to be specific to *TZF1*, as the Glc-TOR signaling response in *TZF2* and *TZF3 OE* plants remained unchanged (Supplementary Figure S6C-E). Consistent with the phenotypic changes, the transcript level of *TOR* was reduced in roots of *TZF1 OE* plants in a separate time-course experiment (Figure 3F). Consistently, the expression of cell cycle S-phase marker genes was also reduced in *TZF1 OE* plants (Figure 3G). These results supported the notion that reduced Glc-TOR activity in the *prr579* mutant might be caused, at least in part, by elevated expression of *TZF1*. Hence, we further investigated whether the other TZF family members were also involved in Glc-TOR signaling. We first determined the mRNA levels and found that the abundance of nearly all the *TZFs* was increased in *prr579* mutant (Supplementary Figure S8). Intriguingly, overexpression of *TZF4*, but not *TZF5* and *TZF6*, caused partial reduction of Glc-TOR signaling (Supplementary Figure S9). Consistently, the *tzf1 tzf4* double mutant also caused a moderate enhancement of Glc-TOR signaling phenotypes and a significantly increased transcript level of *TOR* (Figure 4). Furthermore, we investigated the root architecture of *tzf1 tzf4* double mutant. Compared to the WT, both primary root elongation and lateral root number were significantly enhanced by glucose or sucrose in *tzf1 tzf4* double mutant (Supplementary Figure S10). Taken together, these results indicate that the selected TZF members are redundantly involved in Glc-TOR signaling.

### TZF1 acts downstream of PRR proteins in modulating Glc-TOR signaling

**Figure 5.**
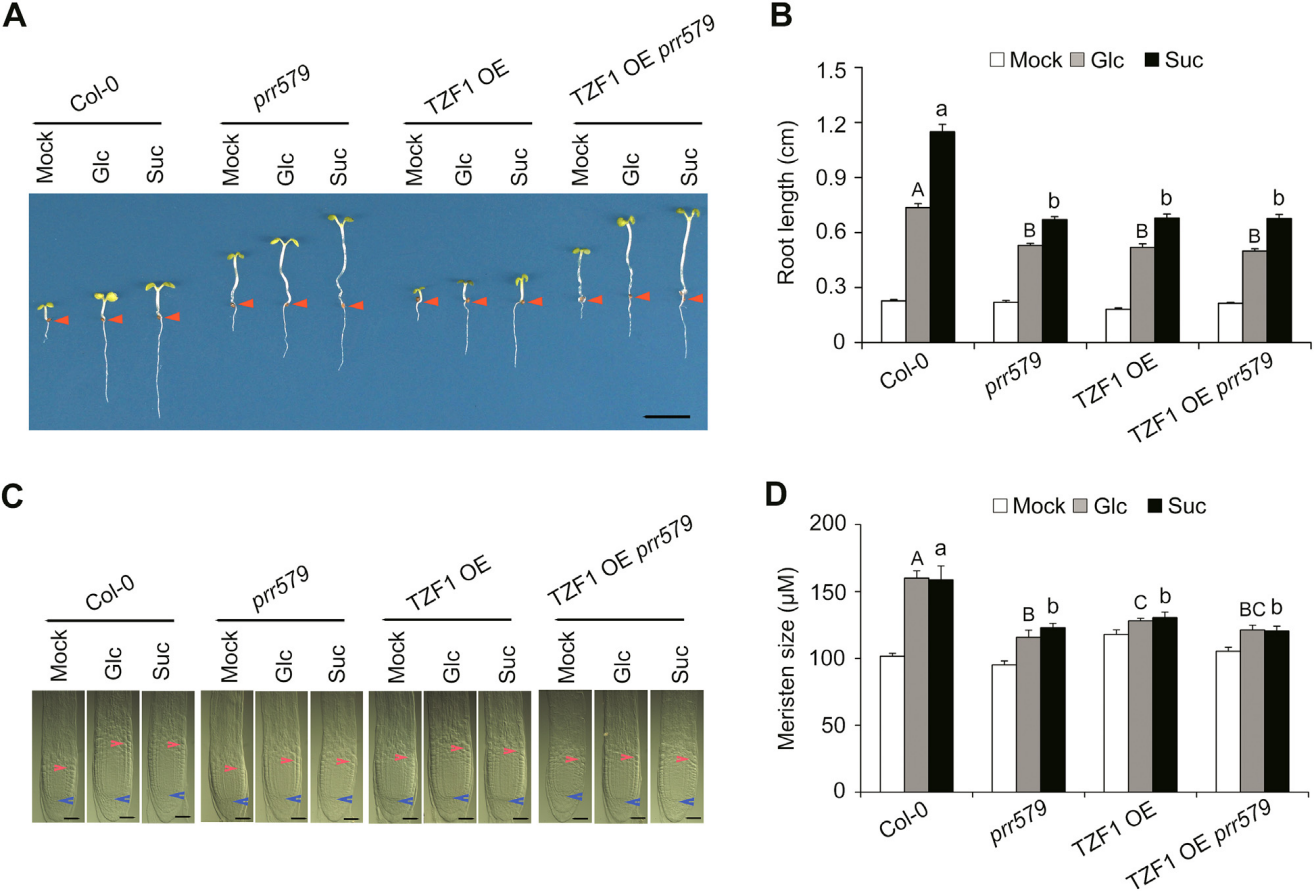
TZF1 acts downstream of PRR proteins to modulate TOR signaling. (**A**) The primary root length reduction of *TZF1 OE prr579* was not additive of *prr579* and *TZF1 OE* upon activation of TOR signaling by sugars. Three-day-old seedlings grown in liquid sugar-free 1/2 MS were treated with 15 mM Glc or Suc for 3 days in weak light (22°C, LD). (Scale bar: 0.5 cm). (**B**) Quantitative analysis of the primary root length in (A). Red arrows indicate the hypocotyl and root junctions. Data represent mean ± s.e.m. of 16 plants. The result was shown from one of three independent experiments with similar results. Root lengths were measured by using ImageJ. (**C**) DIC imaging of root meristem zones. Scale bar: 50 μm. The blue arrow indicates the root quiescent cells, and the red arrow indicate the transition between meristem zone and elongation zone. (**D**) Quantitative analysis of the root meristem size in (**C**). Data represent mean ± s.e.m. of 15 plants. The result was shown from one of three independent experiments with similar results. The root meristem size was measured by using ImageJ. Different letters in (B) and (D) represent the significant difference as *P* < 0.05 by one-way ANOVA with SPSS software. Uppercase letters compare with each other in 15 mM Glc treatment condition, and lowercase letters compare with each other in 15 mM Suc treatment condition.

Because *TZF1* is a direct target gene of PRR proteins, and Glc-TOR signaling was compromised in *TZF1 OE* plant, we reasoned that PRR proteins might modulate Glc-TOR signaling by repressing the expression of *TZF1*. Because neither *TZF1* null mutant nor *TZF1/2/3* RNAi lines showed any obvious defects in Glc-TOR signaling, we crossed the *TZF1 OE* line with the *prr579* mutant to generate *TZF1OE prr579*. Compared to the WT, both the length of primary roots (Figure 5A and B) and the size of root meristems (Figure 5C and D) of *TZF1OE prr579* seedlings were significantly reduced. Evidently, the reduction of Glc-TOR signaling in *TZF1OE prr579* seedlings was not additive, compared to either *TZF1 OE* or *prr579* mutant alone (Figure 5B and D), indicating that TZF1 worked in the same genetic pathway with PRRs. Interestingly, *TZF1OE prr579* still displayed a long hypocotyl phenotype as *prr579* mutant, suggesting that hypocotyl elongation and root meristem activity might be modulated by distinct mechanisms or the role of TZF1 in hypocotyl was masked by other PRRs downstream factors.

Because TZF1 acts downstream of PRRs in mediating Glc-TOR signaling, we further determined if TZF1 could feedback modulate circadian period by crossing the *CCA1pro:LUC* reporter with *TZF1 OE* lines. No discernible changes of circadian period were found using either a bioluminescence assay or RT-qPCR by testing the temporal expression pattern of *CCA1* and *GIGANTEA* (Supplementary Figure S11), indicating that PRRs-TZF1 module mediates circadian outputs without involving in feedback modulation of circadian clock.

### The TZF motif is essential for mediating Glc-TOR signaling

**Figure 6.**
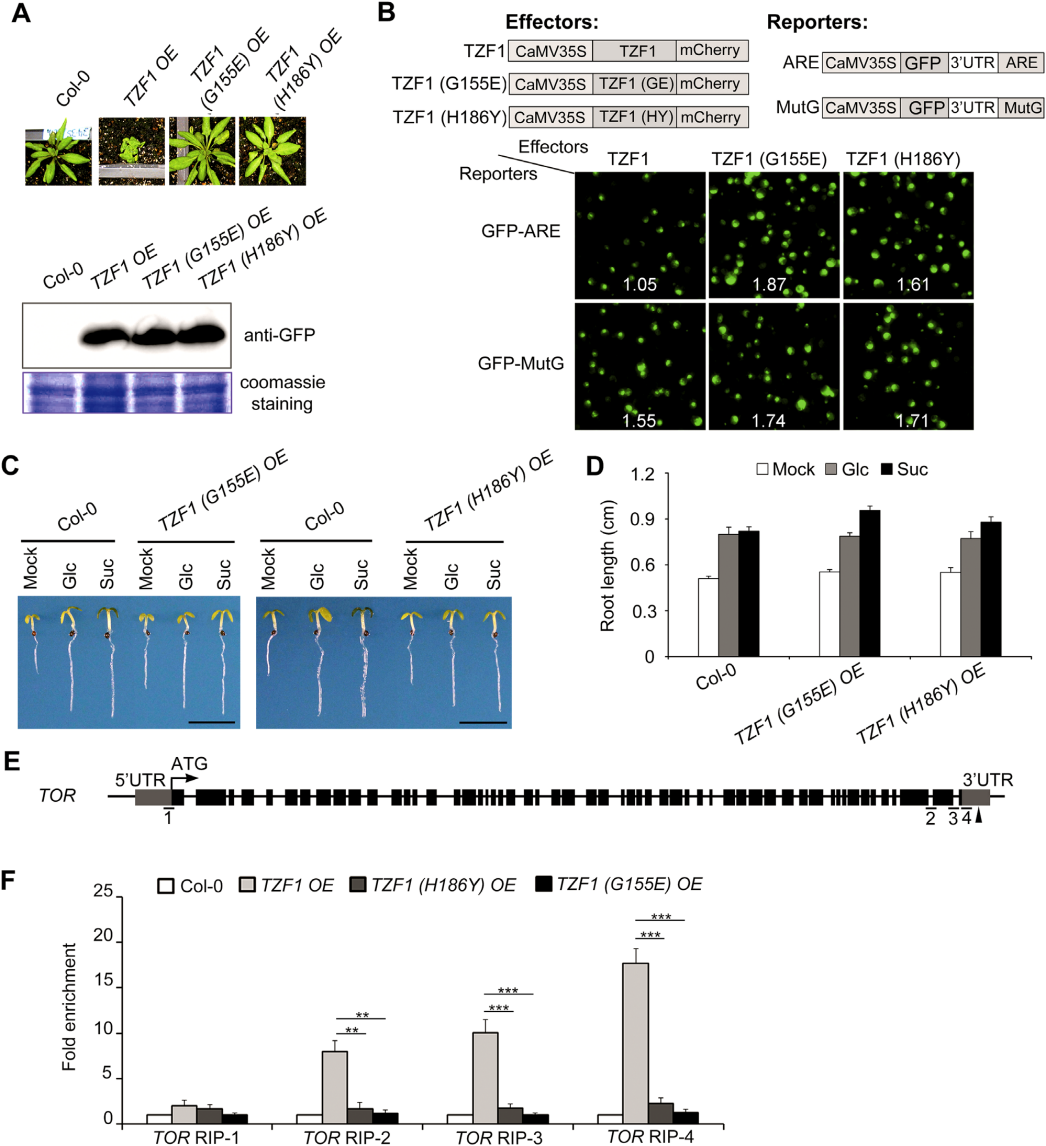
TZF motif is critical for TZF1 to bind *TOR* mRNA and mediate TOR signaling. (**A**) The two intragenic mutations, G155E and H186Y in the transgene *CaMV35S:TZF1-GFP* abolished the *TZF1 OE* phenotypes. Compared to *TZF1 OE* plants, the two EMS point mutants (G155E in inter-ZF region and H186Y in 2^nd^ zinc finger) displayed non-compact WT phenotypes. The expression levels of the two mutant GFP fusion proteins were comparable to that of the WT TZF1-GFP in a Western blot analysis detected by the GFP antibody. (**B**) *TZF1 OE* mutants (G155E and H186Y) were ineffective in triggering mRNA decay. *Arabidopsis* protoplasts were transiently co-expressed with a pair of effector (TZF1 WT or mutant protein) and reporter (GFP fused with ARE or mutated ARE (MutG) at 3′UTR) (see Lin *et al.*, 2011 for detailed methods). WT TZF1 protein triggered GFP-ARE (but not GFP-MutG) reporter gene decay specifically, as indicated by reduced green fluorescence signals (upper left). No differences were observed in any effector-reporter pairs using G155E or H186Y mutant protein. Number in each image indicates the signal ratio of GFP/mCherry derived from the expression of reporter and effector, respectively. (C, D) The *TZF1 (H186Y) OE* and *TZF1 (G155E) OE* lines responded to Glc-TOR signaling normally. Three-day-old seedlings grown in liquid sugar-free 1/2 MS were treated with 15 mM Glc or Suc for 3 days in weak light (22°C, LD). Mock, sugar-free, Glc, glucose; Suc, sucrose. Data represent mean ± s.e.m. of 19 plants. The result was shown from one of three independent experiments with similar results. Root lengths were measured by using ImageJ. Scale bar in (C) is 0.5 cm. (**E**) Diagram depicting the putative TZF1-binding sites in the *TOR*. Black arrowheads indicate the positions of ARE elements, the putative TZF1-binding sites. Fragments underlined with numbers indicate the RIP-qPCR targets. (**F**) RIP–qPCR results indicated a direct binding of TZF1 to *TOR* mRNA. Ten-day-old seedlings grown at 22°C under LD condition was collected at ZT8 for this analysis. Data represent mean ± s.e.m. of three biological replicates. The asterisk indicates significant difference as ** *P* < 0.01 and *** *P* < 0.001 by *t*-test.

Because the TZF motif of TZF1 protein is required for both RNA targeting and turnover (26), we employed a mutant approach to determine if the TZF motif is also essential for Glc-TOR signaling. The *TZF1(G155E) OE* and *TZF1(H186Y) OE* mutant alleles were obtained from an ethyl methanesulfonate (EMS) mutagenesis suppressor screen aiming to identify any intra- or extra-genic mutations that could revert the typical compact and late flowering phenotypes of *TZF1 OE* plants (Supplementary Figure S12A). The morphology of *TZF1(G155E) OE* and *TZF1(H186Y) OE* was indistinguishable from the WT. The mutations in each of the two intragenic mutants, TZF1(G155E) and TZF1(H186Y), were located in the conserved TZF motif of the transgene *CaMV35S:TZF1-GFP* (Figure 6A, Supplementary Figures S12B and S12C). The triple backcrossed homozygous mutant plants had TZF1 protein levels comparable to that in the normal *TZF1 OE* plants, ruling out the possibility of phenotypic reversion caused by gene silencing (Figure 6A). Compared to the TZF1 protein, TZF1(G155E) and TZF1(H186Y) were unable to efficiently trigger the turnover of AU-rich element-containing mRNAs (Figure 6B). Remarkably, neither *TZF1(G155E)* nor *TZF1(H186Y) OE* plants displayed any abnormal sensitivity to Glc-TOR signaling and the reduced TOR transcript levels (Figure 6C and D, Supplementary Figure S13), suggesting that a functional TZF motif is required for mediating TOR signaling.

TZF1 is localized in Processing bodies (P-bodies) and stress granules (SGs) (25). TZF1 specifically binds AU-rich elements (ARE) of mRNA and triggers degradation of corresponding mRNAs (Figure 6B) (26). Hence, we were prompted to investigate if reduced *TOR* mRNA in *TZF1 OE* lines was correlated with its binding to *TOR* mRNA, especially given that 3′-UTR of *TOR* containing at least three AU-rich elements. RNA-immunoprecipitation-RT-qPCR assay (RIP-RT-qPCR) was conducted to determine the levels of TZF1 binding in different regions of *TOR* mRNA (Figure 6E). A significant enrichment of TOR amplicons in *TZF1 OE* line was detected, but not in *TZF1(H186Y) OE* or *TZF1(G155E) OE* line (Figure 6F). Consistently, the transcript abundance of *TOR* was not significantly reduced in *TZF1(H186Y) OE* and *TZF1(G155E) OE* (Supplementary Figure S13). Because TZF1 and TZF4 were redundantly involved in Glc-TOR signaling (Figure 4 and Supplementary Figure S9), we examined if TZF4 could also bind *TOR* mRNA. Results showed that TZF4 could bind *TOR* mRNA as well (Supplementary Figure S14), consistent with its redundant role with TZF1. Therefore, we propose that TZF1 and TZF4 act as negative regulators of TOR signaling, likely by direct binding to the 3′-UTR and triggering *TOR* mRNA degradation in P-bodies.

### Abnormal root architecture in *TZF1 OE* lines caused by compromised Glc-TOR signaling

**Figure 7.**
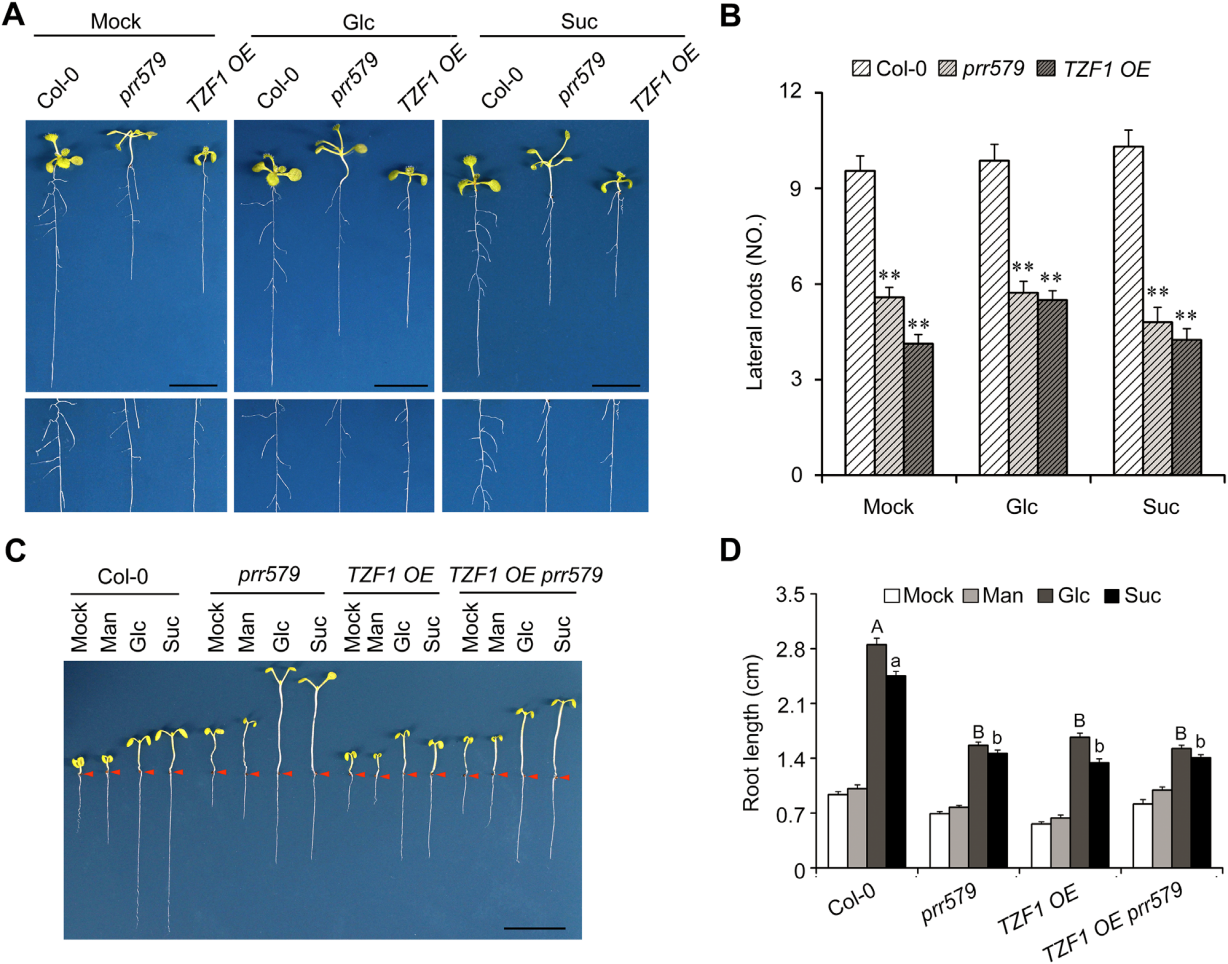
Diminished TOR-signaling affects root architecture in *TZF1 OE* line and *prr579* mutant. (**A**) Reduced lateral root number in *prr579* mutant and *TZF1 OE* line. The lower panel is a close-up view of the upper panel. The seedlings were grown on solid MS plates with 1% sucrose at 22°C under LD condition for 14 days. (**B**) Quantitative analysis of lateral root number per plant as shown in (A). Data represent mean ± s.e.m. of 15 plants. The result was shown from one of three independent experiments with similar results. The asterisk indicates significant difference as ** *P* < 0.01 by *t*-test. (**C**) Primary root elongation was reduced in both *prr579* mutant and *TZF1 OE* line. The seedlings were grown on MS plates for 14 days in weak light (22°C, LD). Scale bar: 1 cm. Red arrows indicate the hypocotyl and root junctions. (**D**) Quantitative analysis of primary root length. The root lengths were measured by using ImageJ. Mock, sugar-free; Man, mannitol; Glc, glucose; Suc, sucrose. Data represent mean ± s.e.m. of 15 plants. The result was shown from one of three independent experiments with similar results. Different letters represent significant difference as *P* < 0.05 by one-way ANOVA with SPSS software. Uppercase letters compare with each other in 15 mM Glc treatment condition, and lowercase letters compare with each other in 15 mM Suc treatment condition.

Blocking Glc-TOR signaling either by rapamycin treatment or utilizing inducible promoter to drive the expression of an RNA interference construct to reduce *TOR* expression shortens primary roots and reduces lateral root number (43), which were also found in the *prr579* mutant (7). Since TZF1 potentially acted downstream of PRR proteins in Glc-TOR signaling, we further examined if diminished TOR signaling could affect root architecture in *TZF1 OE* lines. Compared to the WT, both primary root length and lateral root number were significantly reduced in *TZF1 OE* lines, to the levels that were comparable to that in *prr579* (Figure 7A and B). The shortened primary root phenotype was further investigated under weak light to limit photosynthesis. For the WT, primary root elongation was significantly enhanced by glucose or sucrose, but not by an inactive carbon source, mannitol. By contrast, the root elongation in both the *prr579* mutant and the *TZF1 OE* line was significantly reduced (Figure 7C and D), likely due to reduced Glc-TOR signaling. The phenotypes of *TZF1 OE prr579* were similar to either *TZF1 OE* or *prr579* (Figure 7C and D), further supporting the idea that PRRs and TZF1 orchestrate Glc- TOR signaling in the same pathway to affect root architecture.

## DISCUSSION

**Figure 8.**
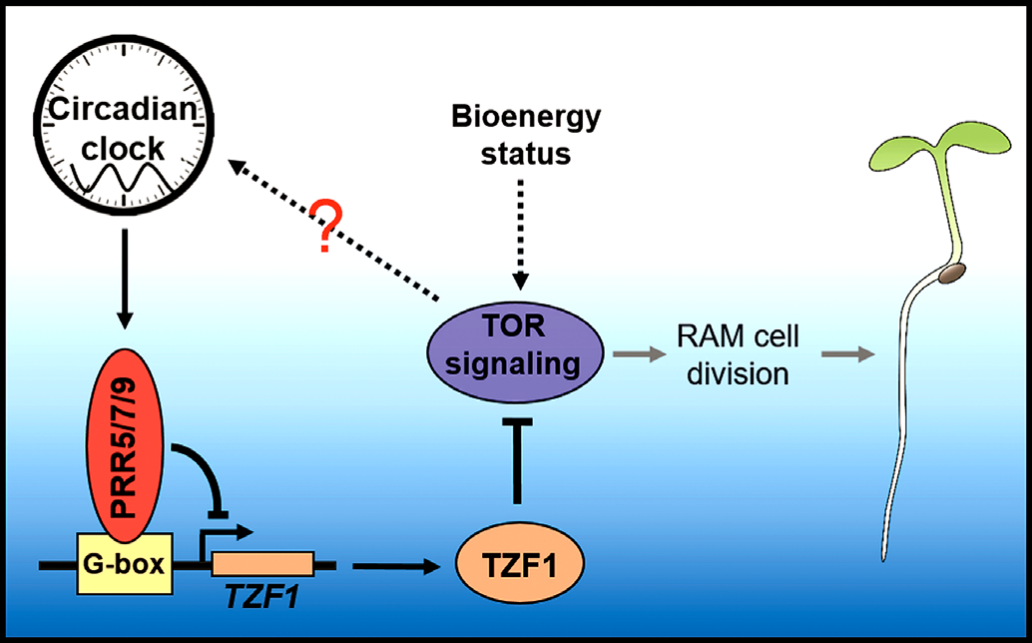
Proposed model of a novel plant circadian clock output mediating TOR signaling through PRRs-TZF1-TOR module. The underlying mechanism by which TOR feedback affects circadian clock remains to be determined.

Although circadian clock has profound impacts on numerous plant processes, whether and how it influences cell proliferation remains largely unknown. Glc-TOR signaling is one of the major sugar signaling pathways to affect root meristem cell proliferation by transducing the endogenous energy status into developmental programs (9). In this study, we have found that the PRR proteins modulate Glc-TOR signaling-mediated root meristem cell proliferation. Moreover, we have further identified that TZF1, a P-body localized RNA binding protein, acts as a direct downstream target of PRR proteins. As a result, PRR proteins modulate Glc-TOR signaling by repressing the expression of *TZF1*. Finally, TZF1 binds 3′-UTR of *TOR* mRNA and attenuates its stability. Taken together, we propose that PRR-TZF-TOR molecular module plays a pivotal role in mediating root meristem cell proliferation (Figure 8).

PRR proteins are core circadian clock components serving as major regulators in mediating a number of circadian outputs, including cold stress response, light signaling, and iron homeostasis (4,5,44). Notably, PRR proteins also play essential roles in mediating endogenous sugar signaling to entrain circadian rhythms. PRRs have also been inferred in feedback regulation of plant sugar signaling via an unknown mechanism (8). Here, we have revealed that Glc-TOR signaling is diminished in the *prr579* mutant. Concievably, because *TOR* mRNA is oscillated (Supplementary Figure S1) and the amplitudes of *TOR* mRNA levels were dampened in *prr579* mutant and *TZF1 OE* plants (Figures 1G and 3F), hence we cannot rule out a possibility that PRR-TZF-TOR pathway is related to circadian regulatory network, directly or indirectly. Future research is warranted to unraveling the relationship among circadian clock, energy homeostasis, and TOR signaling.

The circadian core oscillators function as hubs in modulating a myriad of outputs by controlling the diel expression pattern of key transcription factors participating in distinct biological processes. For example, circadian output mediated CBFs (C-repeat (CRT)/dehydration responsive element (DRE)-binding factors) and PHYTOCHROME-INTERACTING FACTORS (PIFs) are involved in cold stress tolerance and hypocotyl elongation, respectively (5,45,46). *TZF1* belongs to a gene family containing 11 members. Although previously annotated as a putative zinc finger transcription factor, results of various analyses indicate that TZF1 does not possess any transcriptional activities (29). By contrast, TZF1 is involved in post-transcriptional regulation of gene expression by targeting mRNA for turnover (25,26). The integrity of TZF domain is critical for mRNA binding and decay (26). Due to functional redundancy of the *TZF* gene family members, the *TZF1* null mutants were indistinguishable from the WT. By contrast, *TZF1 OE* plants not only displayed distinct developmental and stress response phenotypes (31), but also showed a strong reduction in Glc-TOR signaling response in the present study. Among other *TZF OE* plants tested, we found that *TZF4 OE* plants were also modestly compromised in Glc-TOR signaling. To strengthen the loss-of-function analysis, we constructed the *tzf1 tzf4* double mutant, and found that it displayed an enhanced response to Glc-TOR signaling (Figure 4 and Supplementary Figure S10), supporting the idea that TZF family members are selectively and redundantly involved in Glc-TOR signaling. Moreover, using a forward genetic screen, we identified two critical amino acid mutations in TZF motif that could abolish TZF1′s role in Glc-TOR signaling. Unlike the WT TZF1 protein, the two mutant proteins failed to bind 3′-UTR of *TOR* mRNA. In contrast to the WT TZF1, overexpression of the two *TZF1* mutant alleles did not affect Glc-TOR signaling. Therefore, although the TZF1 null mutant had no distinct phenotypes, the two non-functional TZF1 point-mutant alleles proved to be useful tools in supporting TZF1′s role in Glc-TOR signaling. Taken together, we propose that PRR proteins shape diel expression of *TZF1* and TZF1 protein inturn affects the mRNA accumulation of *TOR* at the post-transcriptional level, hence to temporally mediate a circadian output of Glc-TOR signaling (Figure 8). It is noteworthy that TZF1 does not feedback modulate circadian clock, but only specifically mediates the circadian outputs, resembling some other well-known circadian output regulators (5,45,46).

The root circadian clock has previously been considered as a simplified slave version of shoot circadian clock (47). However, recent reports have demonstrated that circadian clocks within different tissues indeed perform distinct functions (48,49). Differences between the shoot and root clocks are thought to be due to organ-specific sensitivity to light inputs, and the differences are less pronounced in constant darkness (50). Here we used weak light in the presence of sugar to test Glc-TOR signaling sensitivity (9). Under such condition, the exogenous sugar was directly uptaken by roots as energy rather than transported from source to sink. Thus, our findings support the idea that circadian clock plays a role in modulating Glc-TOR signaling-activated root meristem cell proliferation. This agrees with a notion that root growth rate is generally enhanced by sugars, and circadian clock can orchestrate the diel root growth via oscillating sugar signals (51).

TOR signaling has also been shown as required for sugar-induced hypocotyl growth in the dark by stabilizing BRASSINAZOLE-RESISTANT 1 (BZR1), a positive regulator for brassinosteroid response (52). Intriguingly, *prr579* mutant also displayed a long hypocotyl phenotype, which appeared to be independent of Glc-TOR signaling. Moreover, the long hypocotyl phenotype in *prr579* mutant in our study was observed in weak light, not in the dark. Importantly, PRR proteins can directly interact with PIF proteins to sequester or repress PIFs function (53–55). It is conceivable that, in *prr579* mutant, the negative role of PIF proteins in hypocotyl elongation is de-repressed, hence causing the long hypocotyl phenotypes. Compared to *prr579* mutant, *TZF1 OE* plants did display more severe growth retardation phenotypes (31), which was in line with much reduced expression of *TOR* (Figure 3F) and expected phenotypes of Glc-TOR signaling defects (9). Interestingly, *TZF1OE prr579* plants still display long hypocotyl phenotypes, strongly implicating that PRRs’ roles on hypocotyl elongation might be independent of TZF1 or overridden by other PRRs downstream factors, such as PIFs (53–56). Taken together, we propose that PRR proteins modulate hypocotyl cell elongation by affecting transcriptional activities of PIFs, while modulate root cell division activity through PRR-TZF-TOR axis mediated post-transcriptional regulation.

The crosstalk between TOR signaling and circadian clock has been documented in a number of eukaryotic organisms. For instance, BMAL1 (Brain and Muscle Arnt-like protein 1) transcription factor is a core component of the mammalian circadian clock, and its defects cause premature aging and reduced lifespan via negative regulation of mTORC1 signaling in mice (11). Additionally, rhythmic phosphorylation of BMAL1 mediated by ribosomal S6 protein kinase 1 (S6K1), an mTOR-effector kinase, is critical for BMAL1 to be associated with the translational machinery and stimulate protein synthesis (12). Taken together, BMAL1 orchestrates a tight crosstalk between circadian timing and mTOR signaling in mammals. On the other hand, TOR signaling affects the timing of nuclear accumulation of the circadian clock protein TIMELESS to change circadian period in *Drosophila* (57). Moreover, mTOR signaling in the suprachiasmatic nucleus is also engaged in the entrainment and synchronization of the master clock via a translation regulator 4E-BP1 (eukaryotic translational initiation factor 4E binding protein 1) (10). Although our findings here have yet to fully support a crosstalk between circadian clock and TOR signaling in plants, we have provided new insights into a novel mechanism by which PRR-TZF-TOR molecular module shapes root architecture by coordinating clock outputs with cellular metabolism in higher plants. Whether or not TOR signaling can feedback affect circadian rhythms is of a great interest for future investigation.

## Supplementary Material

gkz191_Supplemental_Files
