# Supplementary material for: PRR5, 7 and 9 positively modulate TOR signaling-mediated root cell proliferation by repressing TANDEM ZINC FINGER 1 in Arabidopsis
Source: Nucleic Acids Res. 2019 Mar 20;47(10):5001–15. doi: 10.1093/nar/gkz191 (PMC6547441; doi:10.1093/nar/gkz191)
Supplement: gkz191_Supplemental_Files [file gkz191_supplemental_files.zip › PRR-TZF1-TOR Supporting information.pdf]

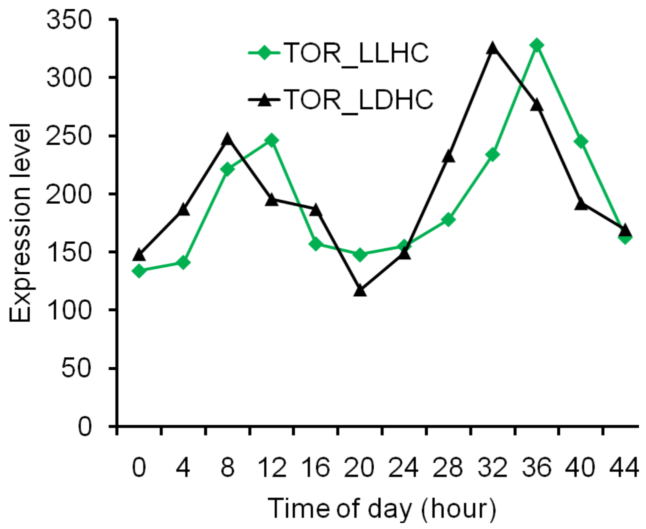

Figure S1. Time-course expression pattern of *TOR* gene. Expression pattern of *TOR* in LD (12-h light /12-h dark) and LL (constant light), respectively. HC represents 12-h hot and 12-h cold. Data were extracted from the DIURNAL database (http://diurnal.mocklerlab.org).

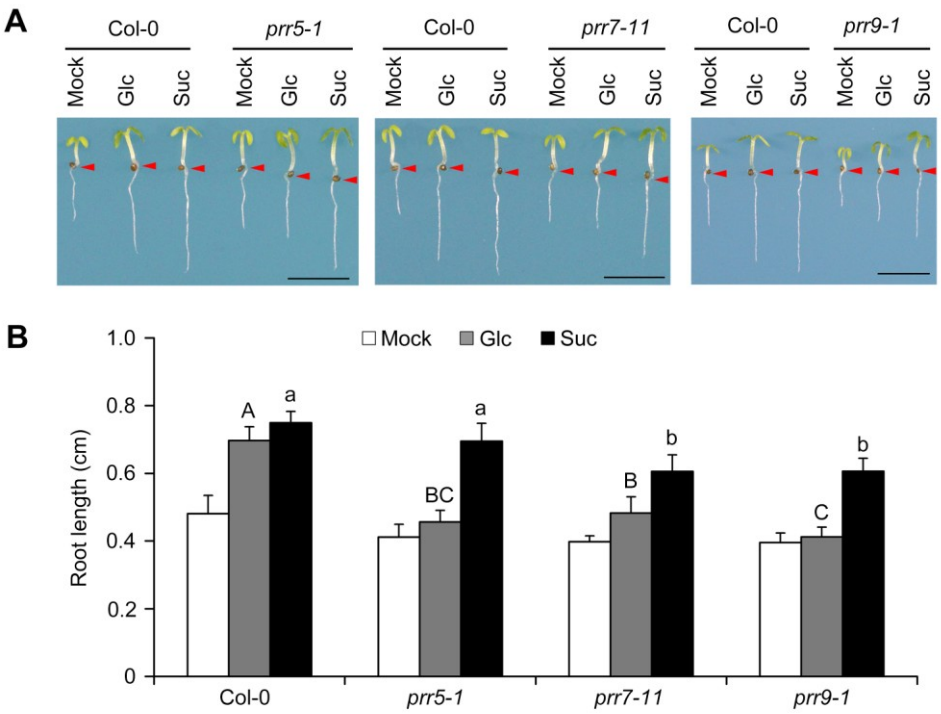

Figure S2. The single mutants of *PRRs* were compromised in Glc-TOR signaling. (**A**) Root growth activation by Glc-TOR signaling is diminished in *prr5*, *prr7*, and *prr9* single mutant (Scale bar: 0.5 cm). The red arrows indicate the hypocotyl and root junctions. (**B**) Quantitative analysis of primary root length. Glc, glucose; Suc, sucrose; Mock, sugar-free. Data represent mean ± s.e.m. of 15 plants. The result was shown from one of the three biological replicates with similar results. Root lengths were measured by using ImageJ. Different letters indicate the significant differences at $p < 0.05$ by one-way ANOVA. Uppercase letters compared with each other in 15 mM Glc treatment condition, and lowercase letters compared with each other in 15 mM Suc treatment condition.

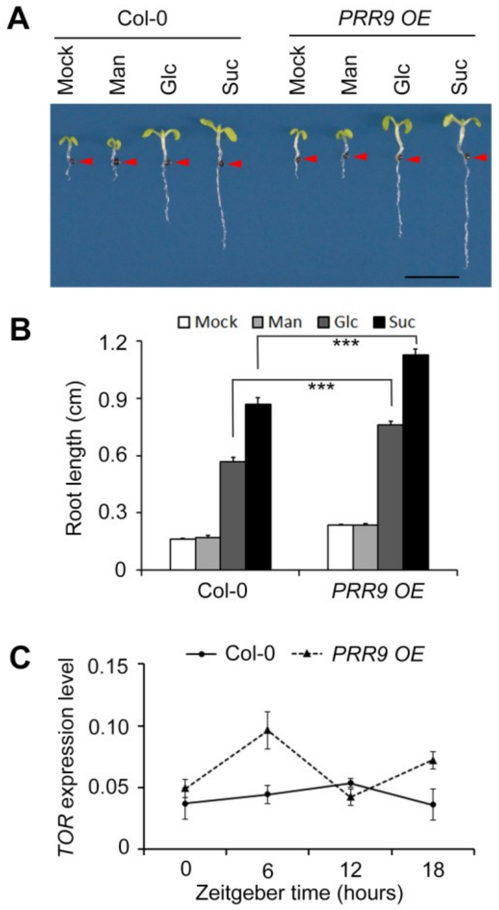

Figure S3. ***PRR9 OE*** **plants were hypersensitive to Glc-TOR signaling.** (**A**) The reactivation of root growth by Glc-TOR signaling was enhanced by overexpression of *PRR9*. Red arrows indicate the hypocotyl and root junctions. DAG, days after germination; D, day; Mock, sugar-free; Man, mannitol; Glc, glucose; Suc, sucrose; (scale bar: 0.5 cm). (**B**) Quantitative analysis of primary root length in (**A**). Data represent mean ± s.e.m. of 15 plants. Three biological replicates were conducted with similar results. Triple asterisks (***) indicate significant difference at $p < 0.001$ by *t*-test. (**C**) Abnormal *TOR* expression in *PRR9 OE*. The total RNA was isolated from the roots grown in half-strength liquid MS medium without sugar in 12-h weak light (13 μmol $m^{-1}s^{-1}$) /12-h dark cycles at 22°C. The gene expression level was normalized by the geometric mean of *ACT2* and *TUB4* expression. Data represent mean ± s.e.m. of three biological replicates.

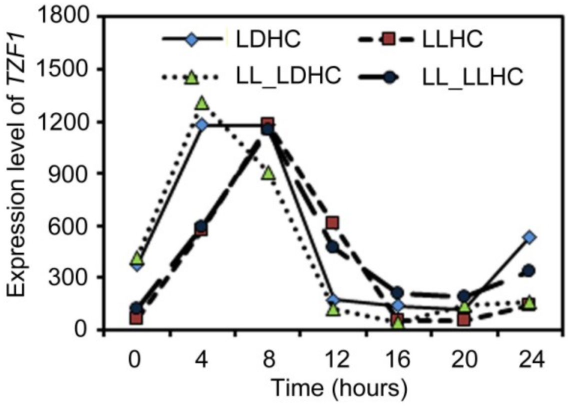

Figure S4. Time-course expression pattern of *TZF1* gene. Expression pattern of *TZF1* in LD (12-h light /12-h dark) and LL (constant light). HC represents 12-h hot and 12-h cold respectively. Data were extracted from the DIURNAL database (http://diurnal.mocklerlab.org).

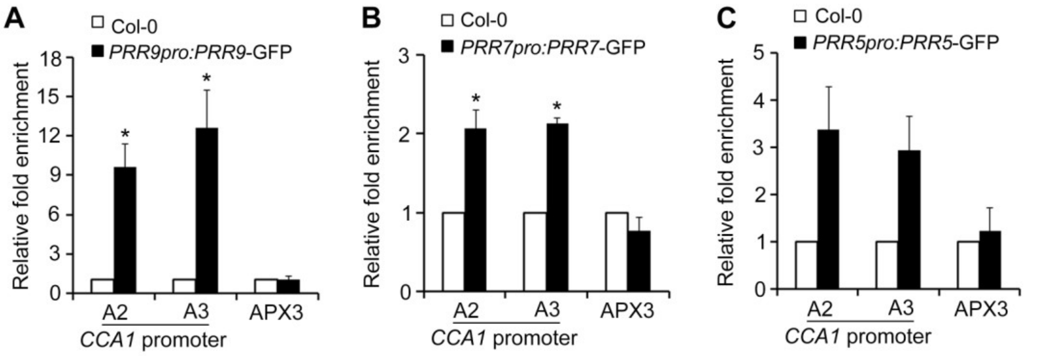

Figure S5. *CCA1* promoter was used as a positive control in ChIP-qPCR analysis. A2 and A3 are the amplicons of *CCA1* promoter. The locations of A2 and A3 in *CCA1*gene are −711/−573 and −589/−443, respectively, relative to the transcript start site. Ten-day-old seedlings of Col-0, *PRR5pro:PRR5-GFP* (ZT10), *PRR7pro:PRR7-GFP* (ZT8), *PRR9pro:PRR9-GFP* (ZT4) grown at 22°C under LD condition were used for chromatin immunoprecipitation analysis. ChIP enrichments were normalized to Col-0. Error bars represent standard error of three biological replicates. The asterisks (*) indicate significant difference relative to Col-0 at $p < 0.05$ by *t*-test.

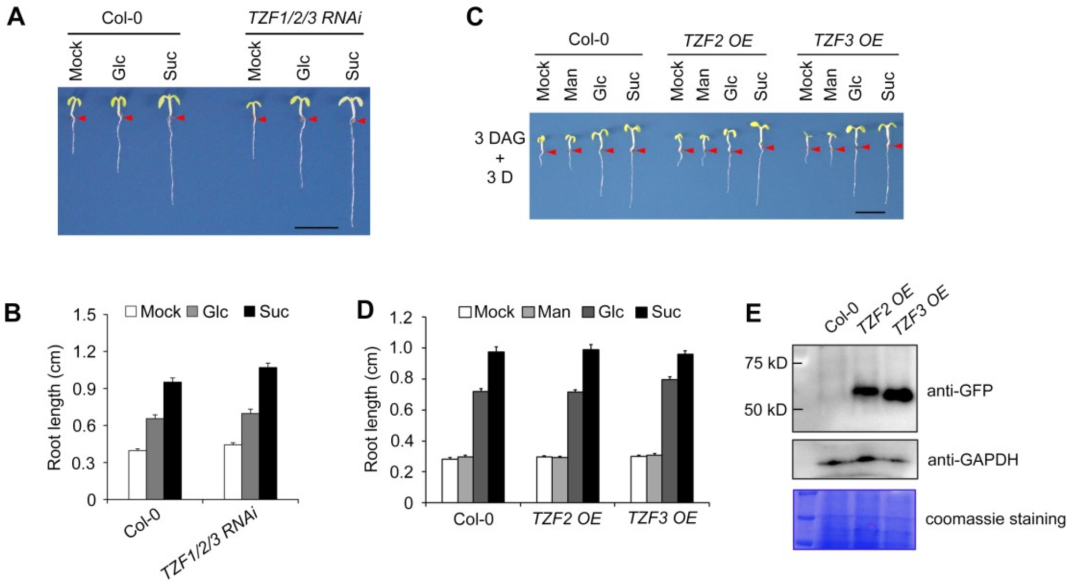

Figure S6. **Glc-TOR signaling was unaffected in either *TZF1/2/3* RNAi or *TZF2* and *TZF3 OE* lines.** (**A**) Glc-TOR signal is unaffected in *TZF1/2/3* RNAi line. (**B**) Quantitative analysis of the primary root length. Glc, glucose; Suc, sucrose; Mock, sugar-free. Data represent mean ± s.e.m. of 15 plants. The result was shown from one of the three biological replicates with similar results. Root lengths were measured by using ImageJ. **(C-D**) TOR signaling is unaffected by *TZF2* or *TZF3* overexpression. Data represent mean ± s.e.m. of 16 plants. The result was shown from one of three biological replicates with similar results. Root lengths were measured by using ImageJ. (**E**) Western blot analysis showing the protein expression of transgene in *TZF2* and *TZF3 OE* lines.

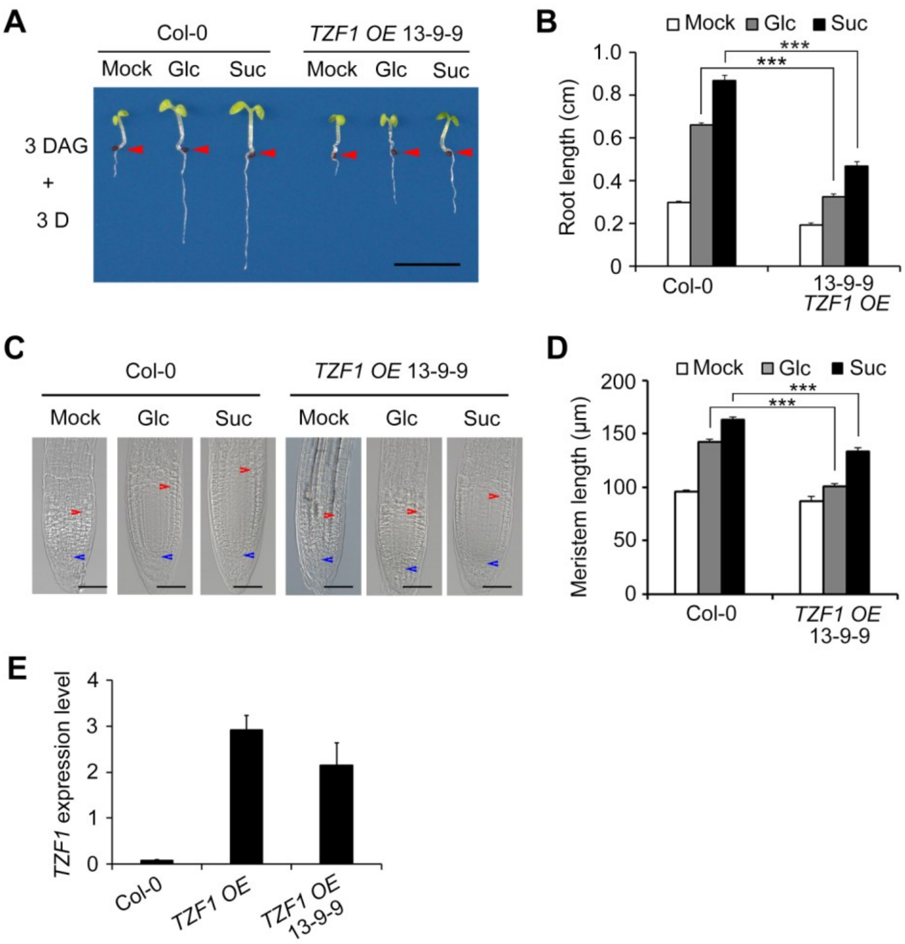

Figure S7. The Glc-TOR signaling response was reduced in a separate *TZF1 OE* line. (**A**) The diminished activation of Glc-TOR signaling in *TZF1 OE* line was evidenced by reduced primary root elongation. Three-day-old seedlings grown in liquid sugar-free 1/2 MS were treated with 15 mM Glc or Suc for 3 days in weak light (22 °C, LD). Red arrows indicate the hypocotyl and root junctions. DAG, days after germination; D, day; Mock, sugar-free; Glc, glucose; Suc, sucrose; (scale bar: 0.5 cm). The line 13-9-9 of *TZF1 OE* is an additional transgenic line. (**B**) Quantitative analysis of primary root length in (**A**). Data represent mean ± s.e.m. of 15 plants. The result was shown from one of three independent experiments with similar results. Root lengths were measured by using ImageJ. (**C**) DIC imaging of root meristem zones. Scale bar: 50 µm. The blue arrows indicate the root quiescent cells, and the red arrows indicate the transition between meristem zone and elongation zone. (**D**) Quantitative analysis of the root meristem size in (**C**). Data represent mean ± s.e.m. of 16 plants. The result was shown from one of three independent experiments with similar results.

The root meristem size was measured by using ImageJ. Triple asterisks (***) in (**B**) and (**D**) indicate significant difference at $p < 0.001$ by *t*-test. (**E**) *TZF1* expression level in two independent transgenic lines. The gene expression level was normalized by the geometric mean of *ACT2* and *TUB4* expression. Data represent mean ± s.e.m. of three biological replicates.

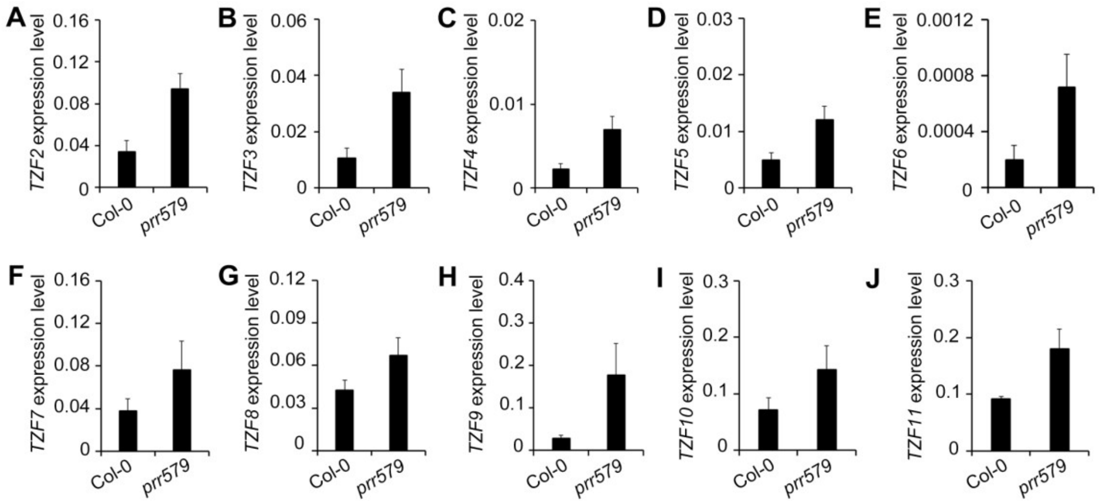

Figure S8. Expression of *TZF* family members in *prr579* mutant and Col-0. The corresponding gene locus IDs are: *At2g19810* (*TZF2*), *At4g29190* (*TZF3*), *At1g03790* (*TZF4*), *At4g44260* (*TZF5*), *At5g07500* (*TZF6*), *At2g41900* (*TZF7*), *At5g12850* (*TZF8*), *At5g58620* (*TZF9*), *At2g40140* (*TZF10*), *At3g55980* (*TZF11*). The total RNA was isolated from the roots of 6-day-old seedlings grown in liquid 1/2 MS medium without sugar in 12-h weak light (13 μmol $m^{-1}s^{-1}$) /12-h dark cycles at 22°C. The gene expression level was normalized by the geometric mean of *ACT2* and *TUB4* expression. Data represent mean ± s.e.m. of three biological replicates.

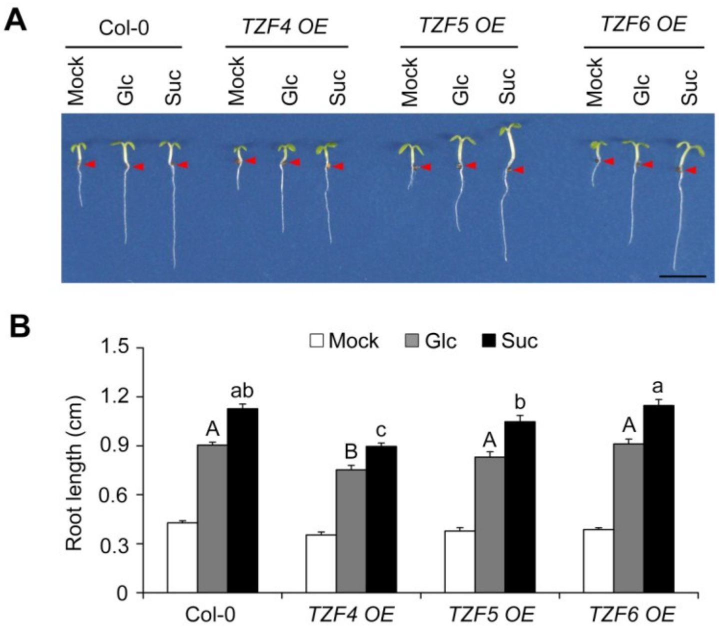

Figure S9. Glc-TOR signaling was marginally affected in *TZF4 OE*, *TZF5 OE*, and *TZF6 OE* lines. (**A**) Glc-TOR signaling was slightly diminished in *TZF4 OE* line, but negligible in *TZF5 OE* and *TZF6 OE* lines. Red arrows indicate the hypocotyl and root junctions. (**B**) Quantitative analysis of the primary root length. Glc, glucose; Suc, sucrose; Mock, sugar-free. Data represent mean ± s.e.m. of 15 plants. The result was shown from one of the three biological replicates with similar results. Root lengths were measured by using ImageJ. Different letters indicate significant differences at $p < 0.05$ by one-way ANOVA. Uppercase letters compared with each other in 15 mM Glc treatment condition, and lowercase letters compared with each other in 15 mM Suc treatment condition.

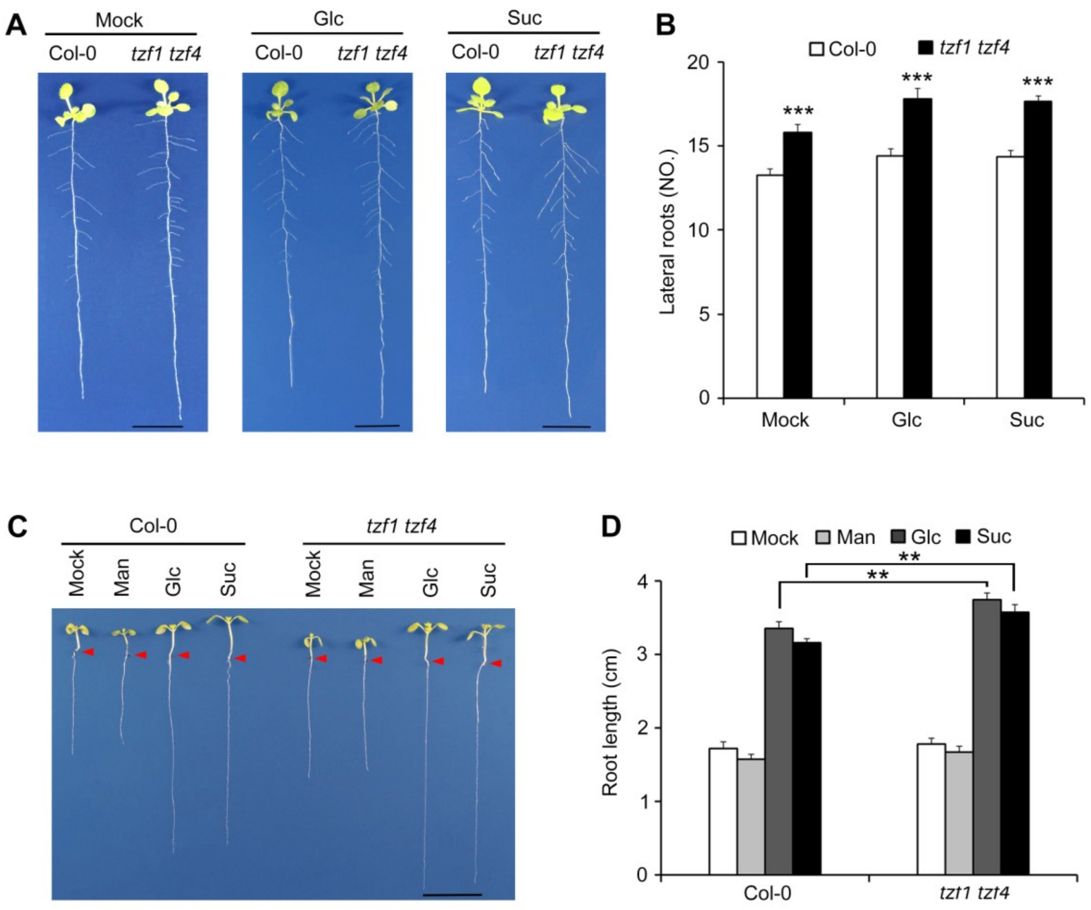

Figure S10. Glc-TOR signaling was synergistically enhanced in *tzf1 tzf4* mutant. (**A**) Increased lateral root number in *tzf1 tzf4* mutant. The seedlings were grown on solid MS plates with 1% glucose or sucrose at 22°C under LD condition for 14 days. (**B**) Quantitative analysis of lateral root number per plant as shown in (**A**). Data represent mean ± s.e.m. of 15 plants. The result was shown from one of three independent experiments with similar results. The asterisk indicates significant difference as *** $p < 0.001$ by *t*-test. (**C**) Primary root elongation was increased in *tzf1 tzf4* mutant. The seedlings were grown on MS plates for 14 days in weak light (22 °C, LD). Scale bar: 1 cm. Red arrows indicate the hypocotyl and root junctions. (**D**) Quantitative analysis of primary root length. The root lengths were measured by using ImageJ. Mock, sugar-free; Man, mannitol; Glc, glucose; Suc, sucrose. Data represent mean ± s.e.m. of 15 plants. The result was shown from one of three independent experiments with similar results. The asterisk indicates significant difference as ** $p < 0.01$ by *t*-test.

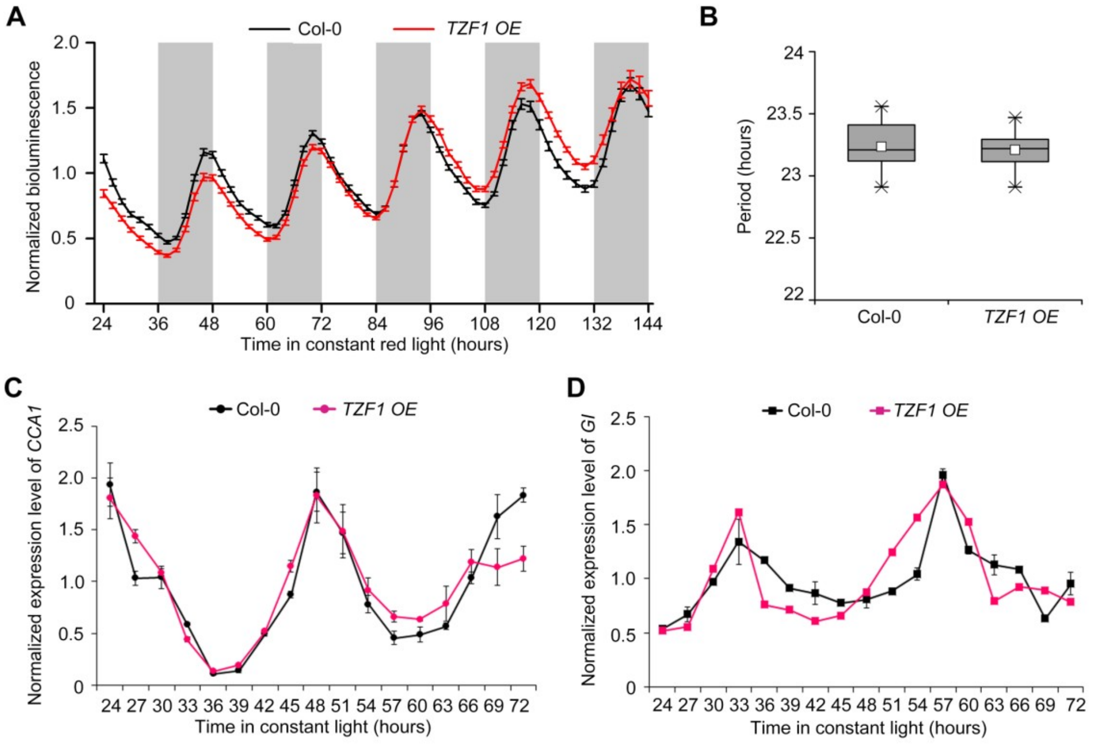

Figure S11. Circadian rhythm was unchanged in *TZF1 OE* line. (**A**) The circadian rhythm was recorded by using *CCA1pro:LUC* reporter under constant red light. (**B**) Period length estimation of (**A**). Data represent mean ± s.e.m. of 18 plants. Horizontal lines are medians, box edges are interquartile ranges and asterisks are minima and maxima. Two biological replicates were conducted. (**C**-**D**) Time-course expression patterns of *CCA1* (**C**) and *GIGANTEA* (**D**) analyzed by RT-qPCR using Col-0 and *TZF1 OE* line under constant light. Data represent mean ± s.e.m. of two biological replicates. The gene expression levels were normalized by *ACT2* expression.

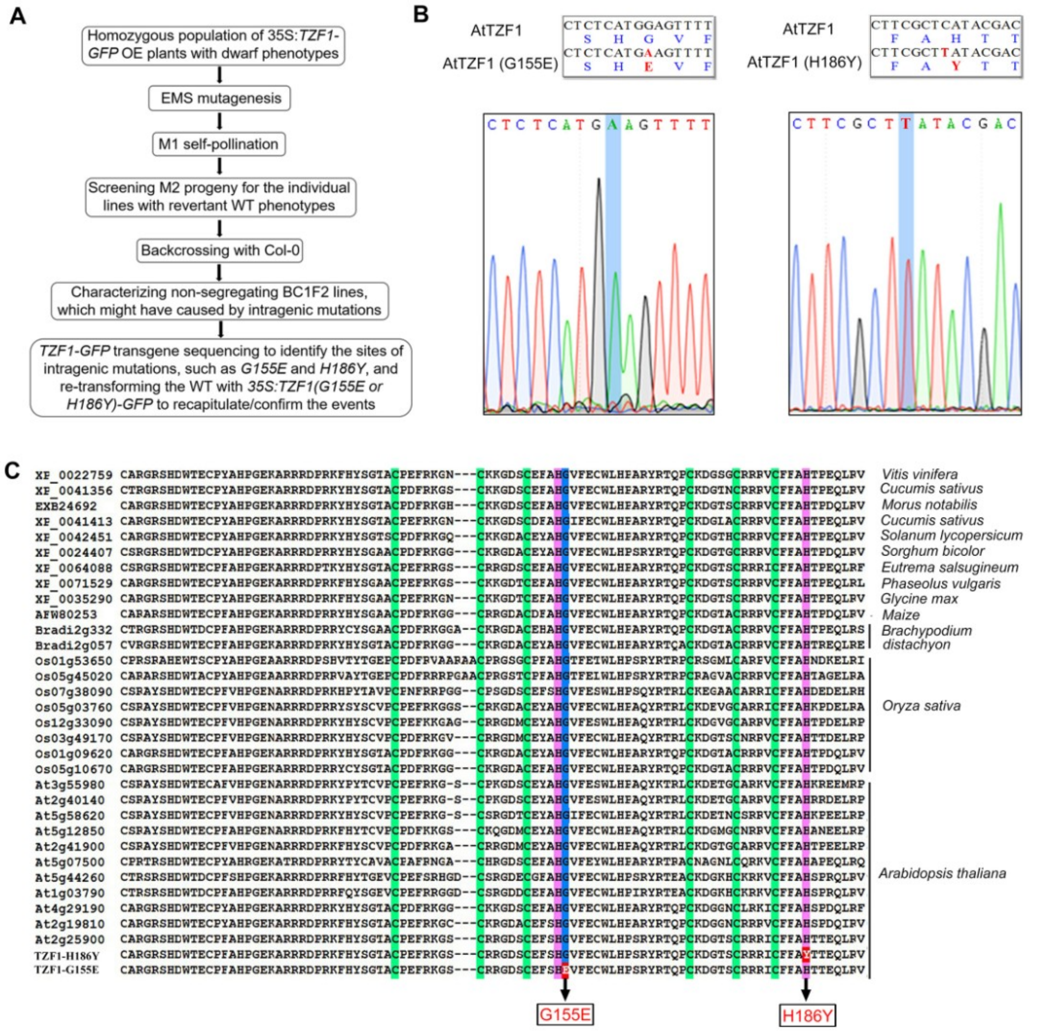

Figure S12. The identification and characterization of *TZF1 (G155E) OE* and *TZF1 (H186Y) OE* mutants. (**A**) Flow chart depicts the processes of genetic screen to obtain *TZF1 (G155E) OE* and *TZF1 (H186Y) OE* mutants. (**B**) The point mutations in *CaMV35S:TZF1-GFP* transgene were identified by Sanger sequencing. The point mutations were highlighted in red in the upper panel, and by the shade in the histograms shown below. (**C**) Alignment of the conserved TZF motifs from selected higher plant species. Arrow indicates the location of G155E and H186Y mutation, respectively.

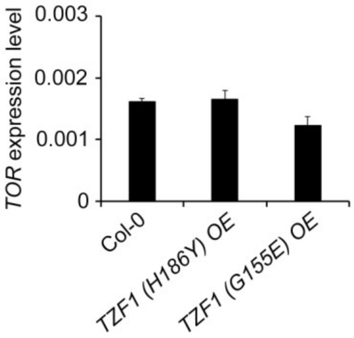

Figure S13. *TOR* expression was not significantly reduced in *TZF1 (H186Y) OE* and *TZF1 (G155E)* lines. Data represent mean ± s.e.m. of three biological technical replicates. The gene expression levels were normalized by the geometric mean of *ACT2* and *TUB4* expression.

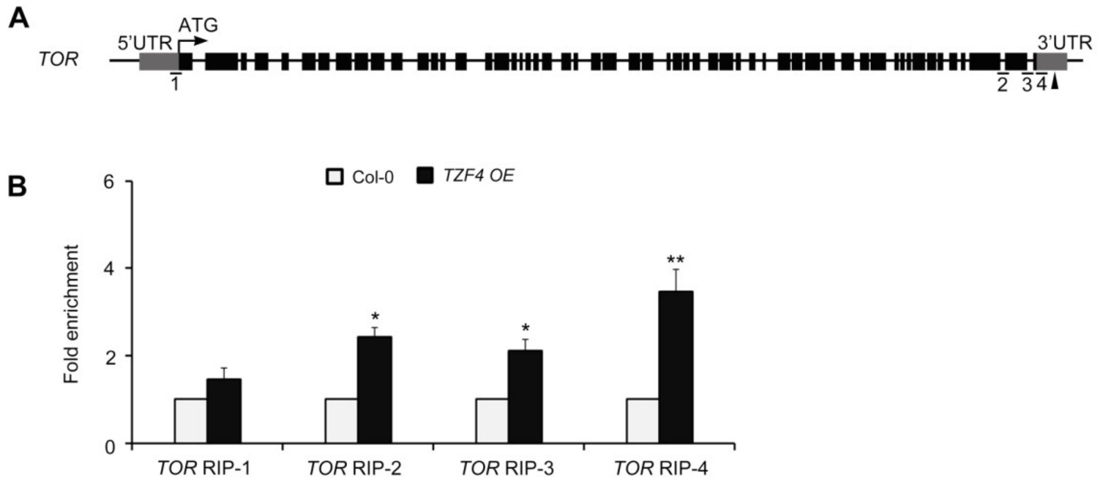

Figure S14. TZF4 protein could bind *TOR* mRNA. (A) Diagram depicting the *TOR* mRNA regions (1 to 4) detected by RIP-qPCR analysis. (B) RIP–qPCR results demonstrated that TZF4 could bind *TOR* mRNA. Ten-day-old seedlings of Col-0 and *TZF4 OE* grown under LD condition were harvested at ZT0. Error bars represent standard error from three biological replicates. The RIP values were normalized to Col-0. The asterisks indicate significant difference at $p < 0.05$ (*), and $p < 0.01$ (**) by *t*-test.

Table S1: Primers used in this study

Primer name	Sequence (5' to 3')
**Primers for RT-qPCR**	
TOR-F	CCGTGGTAATCCTAAGGAGGG
TOR-R	GCTTCACCACTAAGATCACGAA
TZF1-F	TCTCGACTCCTTCGCTTCTCTCTT
TZF1-R	TCGTCGCCACTTGATGAGTCT
GI-F	AGCAGTGGTCGACGGTTTATC
GI-R	ATGGGTATGGAGCTTTGGTTC
CCA1-F	CCTTTTACAAACACCGGCTCTT
CCA1-R	AATCGGGAGGCCAAAATGA
ACT2-F	GCTGAGAGATTCAGATGCCCA
ACT2-R	GTGGATTCCAGCAGCTTCCAT
TUB4-F	AGGGAAACGAAGACAGCAAG
TUB4-R	GCTCGCTAATCCTACCTTTGG
MCM3-F	CTTCGCCACAAGCGAGATTTTATCC
MCM3-R	TGGCTGCGTCACAAAATGACTG
MCM5-F	CAATTCGCCAGCCTTATATCCGAGT
MCM5-R	GGAGCGATCTTGGTGCAAATGTTC
MCM7-F	GCCGACGCTAATGGCAGATCTAA
MCM7-R	GCGGCGGAGAAAATTGAAACATATC
ORC2-F	TGGGTGGGGCGAGTAAGCGT
ORC2-R	AGGCCAAAGCCACACCTGAGC
ORC6-F	CGCCGCCACTAGGTTGCAGATTA
ORC6-R	ACAGCCAAATTGAACCGCCAATTC
CDC6-F	ATGCCTGCAATCGCCGGACC
CDC6-R	GGCAACACCACCGTCGCTGA
ETG1-F	CCCACGCCTCCATTGTCTTATCC
ETG1-R	GAACTGTGCGGCAATGTGATCATTC
PCNA1-F	CCTGATGCTGAGTACCACTCAATCG
PCNA1-R	TGAGCACAATGTTAGCGGTTCCA
TZF2-F	ATCGGAGAATCTCATCGTGG
TZF2-R	TTCCGTAAATATCAGACACCGT
TZF3-F	CGGGTCTTGTTCGATAAACG
TZF3-R	GAATAAAGGATTGTTACGAGGAAAT
TZF4-F	TCTGATGATCCTTACGCAAGTG
TZF4-R	AATCAGTCCAATCGTGGCTT
TZF5-F	TCCCCTTCGCGACTATAAAG
TZF5-R	TTCGTACATCCGGAAATGGT
TZF6-F	CGGGTACATTTTAAGCAAACC
TZF6-R	ATCTGAGCCGTAGATCGCA
TZF7-F	GATCAGACCGATTAAACCAGATC
TZF7-R	CATGTCTGTGTTGGTAACAAGATTG

TZF8-F	GTGGTCTTGCTAAGAAGCTGGA
TZF8-R	GCATCATTCATGGGTTTGG
TZF9-F	CCTCAAATTCTCTCTTCTGCTAGA
TZF9-R	CAATGCTCTCAAGACCTTCTTCT
TZF10-F	GGCAGAAATCAGAAGACGGA
TZF10-R	TCTTCGATCTCTCTCTTAAACGATG
TZF11-F	CAGTGGACCAAAGAGCAATCT
TZF11-R	TTCGAGGAGAAGCATTGTTTC
**Primers for ChIP-qPCR**	
Amplicon A (-1460 to -1384 *TZF1*)-F	GGGGAGTAGTCTACGACCTTGAAAG
Amplicon A (-1460 to -1384 *TZF1*)-R	TTACAGTAAGAAAATATTTGGACGG
Amplicon B (-192 to -98 *TZF1*)-F	TTGGAGATTTTTTCTTACACGTGTC
Amplicon B (-192 to -98 *TZF1*)-R	TATGAAAATCTTGACCCTATCCACA
Amplicon C (-54 to +23 *TZF1*)-F	GCAGTTTATATCACAGACACACACC
Amplicon C (-54 to +23 *TZF1*)-R	TTTTTATTTTCGCCGATCATCATTT
Amplicon D (-270 to -58 *APX3*)-F	CTCGTGTGCCGTTTTTTG
Amplicon D (-270 to -58 *APX3*)-R	GTCAAGGATGTGTGTGAAG
A2 (-711 to -573 *CCA1*)-F	GTGAGAATAGCGCGTGTAGTGAAC
A2 (-711 to -573 *CCA1*)-R	GATCGTTAATCTCTCCC
A3 (-589 to -443 *CCA1*)-F	GGGAGAGATTAACGATC
A3 (-589 to -443 *CCA1*)-R	CATTCTTGTCCATCTTAACATCAACTTG
**Primers for RIP-RT-qPCR**	
TOR-RIP-F1	TCTCTCTGCCCAAGTGTTGTT
TOR-RIP-R1	CGACGAGGTAGACATCGCTG
TOR-RIP-F2	GAAGATGAAGATCCCGCTGA
TOR-RIP-R2	CTCCAAGCATATTTACAGCCTG
TOR-RIP-F3	CGAACATGGTTTGTCTGTGAA
TOR-RIP-R3	AGAAAGGGCACCACCCAAC
TOR-RIP-F4	ATGTTGGGTGGTGCCCTTT
TOR-RIP-R4	CGAATTAGTCAAACATAAATGCAAAT
**Primers for *TZF1pro: LUC* construct**	
TZF1pro-F	TGTCAATTTACTCCCATCAGC
TZF1pro-R	TTTTGTTTAGTGAGTCTAGAAGAGAG
**Primers for *TZF1* mutation constructs**	
TZF1-H186Y-F	GAATCTGTTTCTTCGCTTATACGACGGAGCAGTTA
TZF1-H186Y-R	TAACTGCTCCGTCGTATAAGCGAAGAAACAGATTC
TZF1-G155E-F	GTGTGAGTTCTCTCATGAAGTTTTCGAGTGTTGGC
TZF1-G155E-R	GCCAACACTCGAAAACTTCATGAGAGAACTCACAC
